# Influence of Redox Couple on the Performance of ZnO Dye Solar Cells and Minimodules with Benzothiadiazole-Based Photosensitizers

**DOI:** 10.1021/acsaem.2c02609

**Published:** 2022-11-08

**Authors:** Carlos A. Gonzalez-Flores, Dena Pourjafari, Renan Escalante, Esdras J. Canto-Aguilar, Alberto Vega Poot, José Maria Andres Castán, Yann Kervella, Renaud Demadrille, Antonio J. Riquelme, Juan A. Anta, Gerko Oskam

**Affiliations:** † Departamento de Física Aplicada, CINVESTAV-IPN, Antigua Carretera a Progreso km 6, Mérida97310, Yucatán, México; ‡ Área de Química Física, Departamento de Sistemas Físicos, Químicos y Naturales, Universidad Pablo de Olavide, ES-41013Seville, Spain; § Facultad de Ingeniería, Universidad Autónoma de Campeche-Campus V, San Francisco de Campeche, Campeche24085, México; ∥ Université Grenoble Alpes, CEA, CNRS, IRIG-SyMMES, Grenoble38000, France

**Keywords:** microwave-assisted solvothermal synthesis, photoelectrochemistry, organic dyes, recombination impedance, solar minimodules

## Abstract

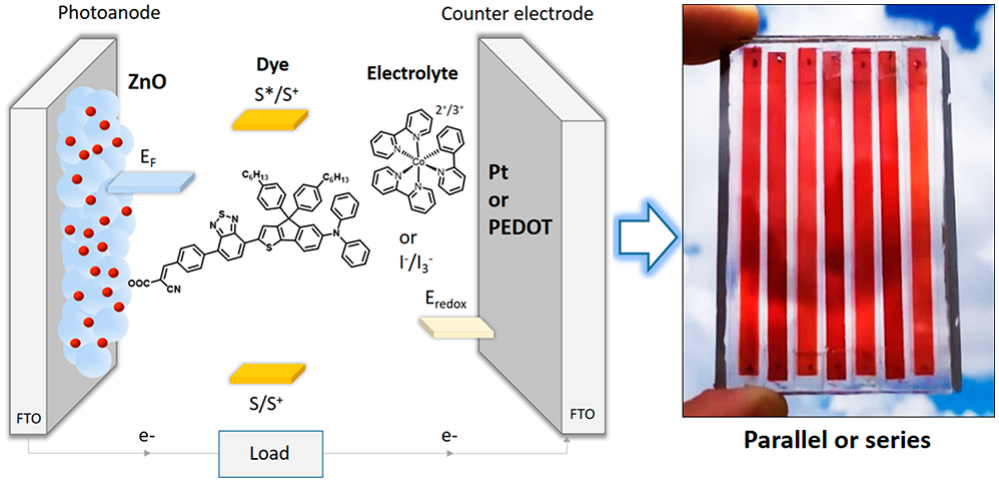

ZnO-based dye-sensitized solar cells exhibit lower efficiencies than TiO_2_-based systems despite advantageous charge transport dynamics and versatility in terms of synthesis methods, which can be primarily ascribed to compatibility issues of ZnO with the dyes and the redox couples originally optimized for TiO_2_. We evaluate the performance of solar cells based on ZnO nanomaterial prepared by microwave-assisted solvothermal synthesis, using three fully organic benzothiadiazole-based dyes YKP-88, YKP-137, and MG-207, and alternative electrolyte solutions with the I^–^/I_3_^–^, Co(bpy)_3_^2+/3+^, and Cu(dmp)_2_^1+/2+^ redox couples. The best cell performance is achieved for the dye–redox couple combination YKP-88 and Co(bpy)_3_^2+/3+^, reaching an average efficiency of 4.7% and 5.0% for the best cell, compared to 3.7% and 3.9% for the I^–^/I_3_^–^ couple with the same dye. Electrical impedance spectroscopy highlights the influence of dye and redox couple chemistry on the balance of recombination and regeneration kinetics. Combined with the effects of the interaction of the redox couple with the ZnO surface, these aspects are shown to determine the solar cell performance. Minimodules based on the best systems in both parallel and series configurations reach 1.5% efficiency for an area of 23.8 cm^2^.

## Introduction

The need for sustainable solutions to meet the ever-increasing world energy demands has led to a drive to develop integrated photovoltaics systems, including product-integrated photovoltaics (PIPV) systems for small energy requirements, such as Internet-of-things (IoT) devices, to automobile (AIPV) and building-integrated systems (BIPV). Dye-sensitized solar cells (DSSCs) are very attractive for this type of applications related to the versatility of the technology, providing systems that may be transparent,^1^ photochromic,^2^ colorful, rigid, or flexible.^3^ In addition, performance is high compared to traditional PV under indoor illumination, which is particularly attractive for IoT applications. On the other hand, DSSC fabrication may be achieved in accordance with green chemistry and sustainable technology principles, using abundant, economic, and nontoxic materials combined with relatively mild fabrication conditions.

However, several factors may limit the DSSC performance. Significant research has been performed to optimize each component of the solar cell such as designing new photoelectrodes,^4−8^ more efficient dyes,^9−11^ and different redox electrolytes,^12−15^ resulting in better insight and understanding of the factors that determine the solar cell performance. One of the essential components of the DSSC is the electron transport layer consisting of a mesoporous semiconducting metal oxide film with large surface area to ensure maximum dye adsorption and, therefore, light harvesting. TiO_2_ has been the material of choice in DSSCs with highest efficiencies reported in the range of 10.9–14.3%, depending on area of the solar cell and the type of dye and redox couple.^16−18^

Current research to fabricate DSSCs tailored to specific applications focuses mainly on novel, organic dyes and alternative redox couples. Fully organic dyes are characterized by a high absorption coefficient, low production cost, and the possibility to change the absorption spectrum and HOMO/LUMO energy levels by incorporation of different electron donor and acceptor groups. Solar cell efficiencies above 10% have been achieved with DSSCs using purely organic dyes.^19,20^ Various redox species such as coordination complexes of cobalt^21−31^ and copper^32−37^ have been proposed to replace the widely employed I^–^/I_3_^–^ redox couple.^38^ The most important advantages include lower absorption, less corrosive nature, and moderate volatility.^39^ A particular goal of using alternative redox complexes is to increase the photovoltage while not significantly decreasing the photocurrent. For indoor lighting conditions, the best efficiencies have been obtained with Cu-based redox complexes.^40^ Changing the redox couple also provides the opportunity to replace the Pt catalyst that is generally deposited onto the counter electrode to accelerate the I_3_^–^ reduction reaction. For Cu-based redox couples, Pt is an insufficient catalyst and has to be replaced, while for the Co-based complexes similar or even better catalytic activity may be achieved with other materials.^41−43^ Alternatives such as gold, graphene, and conductive polymers have been proposed,^44−47^ with advantages in terms of cost, flexibility, and transparency.

The large majority of these novel systems have been developed using TiO_2_ as the electron transporting material. An interesting alternative is ZnO,^48,49^ which has a band gap (3.3 eV) and conduction band edge position that are similar to that of TiO_2_, but this metal oxide is characterized by better electronic transport properties with a bulk electron mobility of 2–3 orders of magnitude higher.^50^ An attractive advantage of ZnO is the great variety of relatively simple and low-temperature synthesis methods and large diversity of nanostructured morphologies, such as nanoparticles,^51,52^ nanosheets,^53,54^ nanotubes,^55^ nanospheres,^56^ and nanowires.^57,58^ Nevertheless, the record efficiency for ZnO-based DSSCs is still significantly lower, with the current record to our knowledge at 8.2%.^59^ Many factors play a role, including the incompatibility of ZnO with many of the dyes originally optimized for TiO_2_, specifically the ruthenium-based coordination complexes that result in high efficiencies and bond to the TiO_2_ surface via carboxylic acid moieties.^60,61^ This is often related to overly acidic conditions of the sensitizing solutions, depending on the dye chemistry, that may etch or erode ZnO nanostructures.^59−61^ Alternative configurations with various ZnO nanostructures, different dyes and redox couples have been reported:^61−65^ for example, a comparison of 1D nanowires and nanoparticulate films for the ZnO/Ru-dye/Co(bpy)_3_^2+/3+^ system reached efficiencies of 5.9% and 4.0%, respectively.^63^ Replacing the Ru-based dye with an organic dye, generally with less acidic bonding groups, resulted in up to 5.7% for 1D ZnO nanowires and 3.6% for nanoparticle ZnO films, respectively.^64^

In this work, we report on the fabrication of dye-sensitized solar cells and scale-up to minimodules of ZnO-based systems, with organic dyes not previously used with this material, and three redox couples. Approximately spherical ZnO nanoparticles have been synthesized by a fast and simple microwave-assisted solvothermal method, and a paste was formulated for film deposition using screen-printing. We study the photovoltaic performance of ZnO photoanodes sensitized with three different organic donor-π-acceptor dyes with a cyanoacrylic bonding moiety (YKP-88, YKP-137, and MG-207) in combination with three redox couples (I^–^/I_3_^–^, Co(bpy)_3_^2+/3+^, and Cu(dmp)_2_^1+/2+^). For each system, electrical impedance spectroscopy (EIS) was performed as a function of light intensity and temperature to provide detailed insights into the mechanisms determining solar cell performance. The highest efficiencies were obtained for the ZnO/YKP-88/Co(bpy)_3_^2+/3+^ system with average efficiency of 4.7%, which is approaching the record efficiencies for ZnO-based DSSCs and higher than the previously reported best efficiency for spherical ZnO nanoparticle-based DSSCs with organic dyes and non-iodide redox couples. Based on these results, the most promising systems were scaled-up to minimodule size (about 24 cm^2^ in active area) in both the parallel and series configurations. We provide a detailed discussion of performance-limiting mechanisms and propose corresponding improvement strategies. This study provides useful guidelines for developing ZnO-based DSSCs by highlighting ZnO/dye/electrolyte interplay.

## Results and Discussion

### Synthesis and Characterization of ZnO, Dyes, and Redox Couples

**Figure 1:**
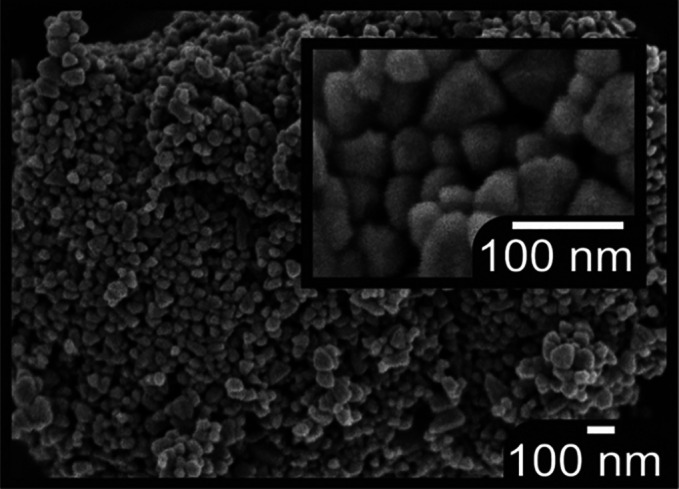
SEM images of a ZnO film deposited by screen printing onto an FTO substrate after sintering at 450 °C. The main image size represents an area of 2.2 μm × 1.6 μm.

ZnO nanoparticles were synthesized using a microwave-assisted solvothermal method using zinc acetate and ethanol in 20 min at 150 °C, and a terpineol-based screen-printing paste was prepared for the deposition of the ZnO photoelectrodes. Figure S1 in the Supporting Information shows the powder X-ray diffraction pattern of the nanomaterial illustrating that pure zincite is formed. Figure 1 shows an SEM image of the screen-printed ZnO film on the FTO substrate after sintering at 450 °C. Uniform nanoparticle morphology is observed with a particle size between 30 and 60 nm, and the homogeneous mesoporous character of the film makes it appropriate for application in the DSSC. Strong interparticle necking is present after sintering, which is essential for efficient electron transport in the nanostructured ZnO film.

**Figure 2:**
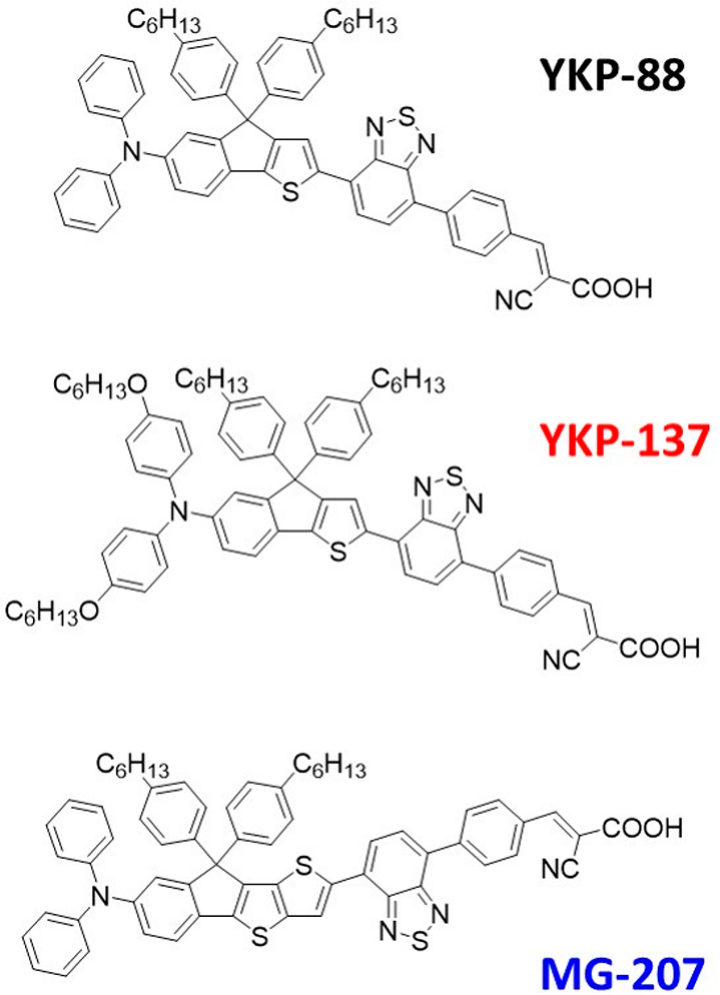
Chemical structures of the YKP-88, YKP-137, and MG-207 organic dyes.

The benzothiadiazole-based organic dyes, shown in Figure 2 and evaluated in this work, were previously applied in TiO_2_-based solar cells, achieving impressive efficiencies of about 9.5%.^65^ YKP-88, YKP-137, and MG-207 follow the original RK-1 dye design^9^ and were modified to shift the absorption of the onset toward longer wavelengths by tuning the push–pull effect. This can be achieved through an increase of the electron-donating strength of the triarylamine moiety or by improving the planarity of the molecules and/or the π-conjugation with the electron-accepting unit. Thus, YKP-137 contains alkoxy groups (C_6_H_13_O) in the electron donor segment, while in MG-207 the thiophene unit flanking the indene ring of YKP-88 is replaced by a thieno[3,2-*b*]thiophene moiety. All three dyes bind to the metal oxide surface via a cyanoacrylic group. Cyclic voltammetry of dye-loaded ZnO films was performed in 0.1 M TBAPF_6_ in acetonitrile to determine the energy level of the highest occupied molecular orbital (HOMO), which is shown in the Supporting Information in Figure S2 and Table S1, where the electrochemical properties measured for the three dyes are specified. Since the LUMO energy is difficult to measure for the dyes adsorbed to ZnO in acetonitrile-based solutions related to a relatively large spurious cathodic current, we used the energy differences between HOMO and LUMO determined in previous work using a DCM (dichloromethane) solution to calculate the energetic position of the LUMO.^65^ It is found that the LUMO energies are essentially identical for the three dyes, indicating that electron injection energetics are expected to not differ significantly.

We evaluated the performance of ZnO-based solar cells using three redox couples: (i) I^–^/I_3_^–^; (ii) Co(bpy)_3_^2+/3+^; (iii) Cu(dmp)_2_^1+/2+^. The goal is to find the optimal combination of properties, which is difficult to predict a priori. In particular, ZnO has been shown to be less sensitive to the effect as band edge determining components compared to TiO_2_, thus limiting the possibility to tune the open circuit potential.^66^ However, different interaction with the dye, related to both the redox couple movement close to the dye-coated electrode surface and the regeneration kinetics, is still expected to greatly affect cell efficiency. In order to determine the relative positions of the dye levels and redox energies, the redox solutions were characterized in detail; Figure S3 and Table S2 in the Supporting Information show the corresponding current–potential curves and the redox potentials obtained, which are in good agreement with previously published values.^61,67^

**Figure 3:**
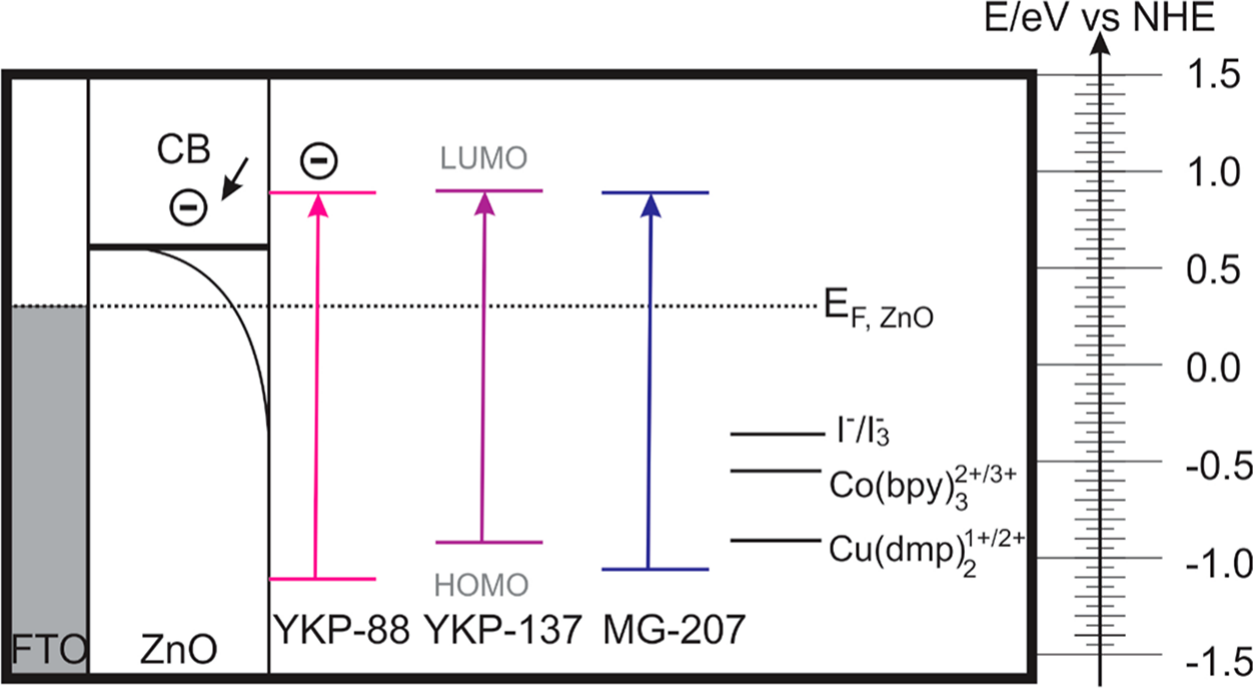
Energy level diagram for the ZnO-based DSSCs using the results from Tables S1 and S2 in the Supporting Information, with the ZnO conduction band edge energy estimated from literature.^48,49,61,67^ Note that the potential vs NHE is given by *V* = −*E*/*e*.

Based on these results, we can construct the energy band diagram in Figure 3 for the different solar cell systems under study, which may give a preliminary idea of the potential of the different systems. It is important to stress that the energy levels are obtained for the independent systems and may not be the same when combined. Furthermore, the conduction band edge energy of ZnO is based on literature values^61,67^ and is prone to depend on dye and redox couple in the actual systems. With these cautionary remarks in mind, we can observe that the excited state energies of all three dyes are essentially the same, which implies that the electron injection efficiency is expected to be similar. On the other hand, a significant effect is expected for the different redox couples, in terms of both open circuit voltage and the short circuit current density. The driving force for dye regeneration is expected to increase in the order I^–^/I_3_^–^ > Co(bpy)_3_^2+/3+^ > Cu(dmp)_2_^1+/2+^, while the open circuit voltage is expected to show the opposite trend. Also, the YKP-137 HOMO energy is about 0.18 eV higher, which may result in critically small driving force, especially with the Cu(dmp)_2_^1+/2+^ redox couple. The balance of these effects is expected to determine the performance of solar cells.

The energy gap between HOMO and LUMO for the YKP-137 dye (1.82 eV) is about 0.15 eV smaller than for the YKP-88 and MG-207 dyes (2.00 and 1.95 eV, respectively), which is also reflected in the measured optical gap and could lead to a higher light harvesting efficiency.^65^ The amount of dye adsorbed was determined by UV–vis measurements after dye desorption, and it was found that using YKP-88 and MG-207 a somewhat better dye coverage was achieved, thus reducing the optical band gap disadvantage. In addition, the dye coverage on ZnO is found to be significantly smaller than observed for YKP-88 on TiO_2_,^20^ indicating a notable difference in the density of adsorption sites on ZnO or adsorption bonding strength. The lower dye coverage for the three dyes on nanostructured ZnO, and in particular for YKP-137, may also significantly affect the recombination kinetics. The UV–vis spectra are shown in Figure S4 and the desorption results are presented in Table S3 in the Supporting Information.

### Solar Cell Performance and Characterization

**Figure 4:**
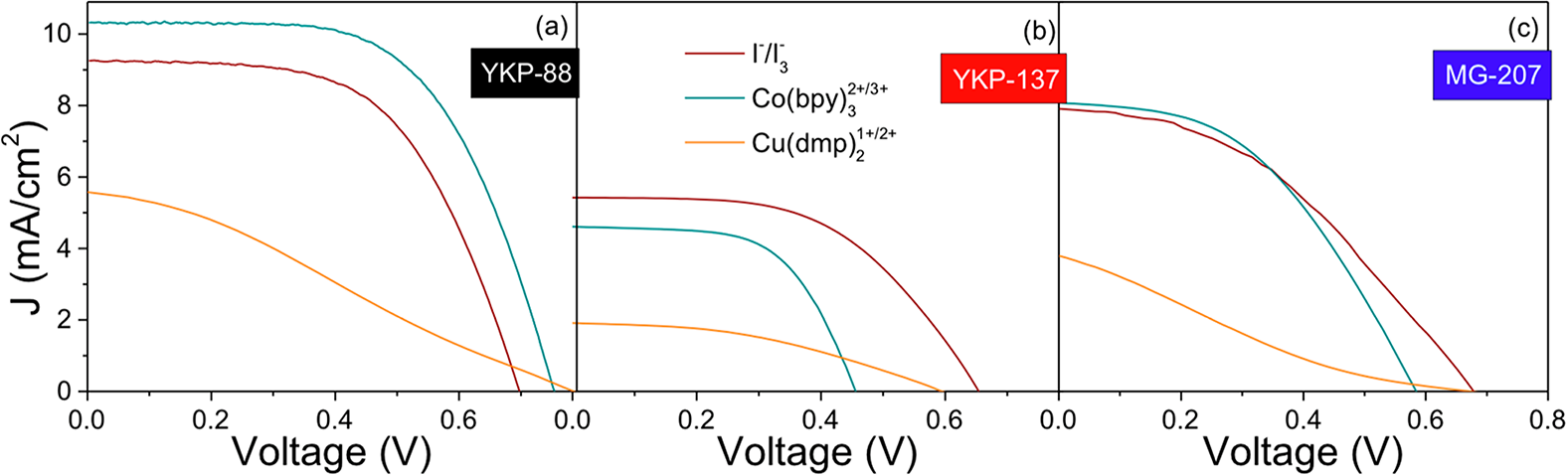
*J*–*V* curves under 1 sun (AM1.5G) illumination of 0.5 cm^2^ ZnO-based solar cells fabricated with (a) YKP-88, (b) YKP-137, and (c) MG-207 and the three different redox electrolyte solutions.

Nine groups of ZnO-based DSSCs were fabricated using the three different organic dyes, YKP-88, YKP-137, and MG-207, and three different redox electrolyte solutions, i.e., I^–^/I_3_^–^, Co(bpy)_3_^2+/3+^, and Cu(dmp)_2_^1+/2+^ in acetonitrile. To ensure appropriate comparison and reproducibility, three cells were manufactured in each group and evaluated. In addition, a separate full set was produced, which showed essentially the same results and tendencies. All devices were fabricated under the same experimental conditions, and parameters such as ZnO film thickness, heat treatment, dye solution concentration, and dye soaking time were the same for all cells. Figure 4 shows current density–potential (*J*–*V*) curves for the best cells in the series, and Table 1 contains the full quantitative details related to the performance of each system, including the photovoltaic conversion efficiency (PCE).

**Table 1:** Solar Cell Performance Parameters under 1 Sun (AM1.5G) Illumination^a^

dye	redox couple	*J*_SC_(mA cm^–2^)	*V*_OC_ (V)	fill factor	PCE (%)
YKP-88	I^–^/I_3_^–^	9.2 ± 0.1	0.69 ± 0.01	0.58 ± 0.03	3.7 ± 0.2
	Co(bpy)_3_^2+/3+^	10.3 ± 0.9	0.75 ± 0.02	0.61 ± 0.05	4.7 ± 0.3
	Cu(dmp)_2_^1+/2+^	5.6 ± 0.6	0.77 ± 0.04	0.29 ± 0.06	1.3 ± 0.1
YKP-137	I^–^/I_3_^–^	5.4 ± 0.1	0.65 ± 0.01	0.54 ± 0.05	1.9 ± 0.1
	Co(bpy)_3_^2+/3+^	4.6 ± 0.2	0.45 ± 0.01	0.60 ± 0.02	1.2 ± 0.1
	Cu(dmp)_2_^1+/2+^	1.9 ± 0.6	0.59 ± 0.01	0.42 ± 0.05	0.5 ± 0.1
MG-207	I^–^/I_3_^–^	7.9 ± 0.1	0.67 ± 0.01	0.41 ± 0.06	2.2 ± 0.3
	Co(bpy)_3_^2+/3+^	8.0 ± 0.1	0.58 ± 0.01	0.45 ± 0.01	2.1 ± 0.1
	Cu(dmp)_2_^1+/2+^	3.8 ± 0.2	0.67 ± 0.01	0.20 ± 0.01	0.5 ± 0.1

a The values represent the average of three cells for each group and the corresponding standard deviation.

Figure 4a shows the *J*–*V* curves of the ZnO-based DSSCs sensitized with YKP-88, illustrating excellent performance with both the I^–^/I_3_^–^ and Co(bpy)_3_^2+/3+^ redox couple, while the curve for the Cu(dmp)_2_^1+/2+^ system is not optimal. The PCE is highest for the YKP-88/Co(bpy)_3_^2+/3+^ system, exhibiting the highest values for *J*_SC_ and a higher *V*_OC_ than observed for the I^–^/I_3_^–^ redox couple. The PCE of the champion cell was 5.0%, and the average efficiency of 4.7% is to our knowledge the highest reported to date for nanoparticulate ZnO-based DSSCs with a purely organic dye and the Co(bpy)_3_^2+/3+^-based redox couple.^64^ The value of *J*_SC_ for the Cu(dmp)_2_^1+/2+^ system is lower than for the other two systems but still reasonable at 5.6 mA cm^–2^, indicating that in this case, electron injection and dye regeneration occur at reasonable efficiency; however, the fill factor is small indicating mechanistic complications and possibly a high series resistance. Although the open circuit potential increases in the order expected, even for the Cu(dmp)_2_^1+/2+^ system, the differences are much smaller than the change in redox potential. From the energy diagram in Figure 3, *V*_OC_ of the DSSC based on the Cu(dmp)_2_^1+/2+^ could be expected to be up to 360 mV higher than the cells based on the Co(bpy)_3_^2+/3+^, and that of cells with Co(bpy)_3_^2+/3+^ was about 190 mV higher than for the I^–^/I_3_^–^ couple. In general, the observation that these shifts are only 80 mV and 60 mV, respectively, implies that either the recombination kinetics are a function of redox couple or a band edge shift occurs, or a combination of both.

Figure 4b shows the *J*–*V* curves for cells prepared with the YKP-137 dye. In general, the performance is much less impressive than for the YKP-88 dye. Interestingly, for this system, *J*_SC_ is highest for the I^–^/I_3_^–^ redox solution and decreases for the Co(bpy)_3_^2+/3+^ and Cu(dmp)_2_^1+/2+^ systems. The very low values for *J*_SC_ observed for the YKP-137/Cu(dmp)_2_^1+/2+^ system cannot be explained by the slightly lower dye coverage but agree with the observations in the energy band diagram in Figure 3, which shows a minimal driving force for dye regeneration. In addition, *V*_OC_ decreases in the order I^–^/I_3_^–^ > Cu(dmp)_2_^1+/2+^ > Co(bpy)_3_^2+/3+^, very different from the observations for the YKP-88 system. These results clearly indicate that the thermodynamically achievable *V*_OC_ is not realized as other processes and mechanisms dominate and that this is particularly true for the YKP-137/Cu(dmp)_2_^1+/2+^ system. The cell with the best performance in this group was fabricated using the I^–^/I_3_^–^ redox couple and showed an average efficiency of 1.9%.

Figure 4c shows the curves for the system using the MG-207 dye. The performance is much better than for the YKP-137 dye, and *J*_SC_ follows the same tendency as for the YKP-88 dye. On the other hand, also for the MG-207 dye the *V*_OC_ is lower for the Co(bpy)_3_^2+/3+^ system, while the fill factor is relatively low for all three systems. Hence, the efficiencies for the systems with the MG-207 dye are generally between those of the YKP-88 and YKP-137 systems, and the highest PCE observed for the I^–^/I_3_^–^ redox couple is 2.2%.

From these results it can be clearly observed that the photovoltaic performance depends on the dye chemistry (see also Figure S5), despite the fact that they all possess a similar π-conjugated backbone and electron acceptor unit, indicating the importance of linking units and modification of the electron donor segments. The lower performance of YKP-137 appears to be related to the presence of the alkoxy groups (C_6_H_13_O) in the electron donor segment of the dye; the somewhat better solar absorption is offset by the lower adsorption coverage. Furthermore, the higher HOMO energy appears critical in causing slower regeneration kinetics. When we compare YKP-88 and MG-207 dyes, it appears that the incorporation of thienothiophene in the chemical structure of the organic dye does not improve the performance of ZnO-based DSSCs. Overall, from the *J*–*V* curves it can be observed that, in general, the performance of YKP-88 dye-sensitized solar cells was the best, in terms of *J*_SC_ and *V*_OC_ and, therefore, PCE.

Comparing different redox couples, the performances achieved with the iodide and cobalt-based redox couples were better than obtained for the copper-based redox couple. Figure S6 in the Supporting Information shows the dependence of *V*_OC_ on temperature for the YKP-88 system with the three redox couples. Extrapolation to 0 K provides a general indication of the maximum attainable *V*_OC_ in the absence of recombination: we find that the intercept for the Co(bpy)_3_^2+/3+^ and I^–^/I_3_^–^ solar cells is about 1.2 eV, significantly smaller than for the Cu(dmp)_2_^1+/2+^ cells of 1.6 eV. These results imply that potentially the *V*_OC_ with the Cu(dmp)_2_^1+/2+^ redox couple may be significantly larger, in accordance with expectations from Figure 3, as long as the balance of recombination and regeneration kinetics is favorable.

When changing the redox couple, it may also be necessary to change the catalyst on the counter electrode. For the I^–^/I_3_^–^ and Co(bpy)_3_^2+/3+^ redox couples, Pt has been shown to work well, although for the latter graphene nanoflakes and Au are also good catalysts that may work better under certain circumstances.^41,42^ For the Cu(dmp)_2_^1+/2+^ system, it has been reported that PEDOT is a superior catalyst for Cu(dmp)_2_^2+^ reduction and that Pt should be avoided.^43^ Our experience presented in Figure S7 confirms this observation: the shape of the *J*–*V* curve of the YKP-88/Cu(dmp)_2_ system improves, and although both *J*_SC_ and *V*_OC_ increase upon replacing the platinum catalyst with PEDOT, especially the fill factor improves. The charge transfer resistance at the counter electrode may significantly affect the fill factor, confirming the better catalytic properties of PEDOT for this specific redox couple. DSSCs with the PEDOT-coated counter electrode reach an efficiency of 2.1% compared to the platinum-catalyzed system of 1.3%; this may be further optimized by improving the PEDOT catalyst. However, the performance is still significantly worse than for the other two redox couples, indicating that other processes also limit the efficiency. In particular, the low dye coverage on the ZnO surface allows relatively easy access of the redox couple to the oxide surface, which is expected to increase chemical interactions at the surface as well as increased recombination kinetics. In addition, charge transfer processes at ZnO surfaces have been shown to be generally faster than at TiO_2_, which exacerbates these effects.^48,49,60,66^ The recombination dynamics can be evaluated using small-signal perturbation methods as a function of the frequency, such as electrical impedance spectroscopy.

### Electrical Impedance Spectroscopy

**Figure 5:**
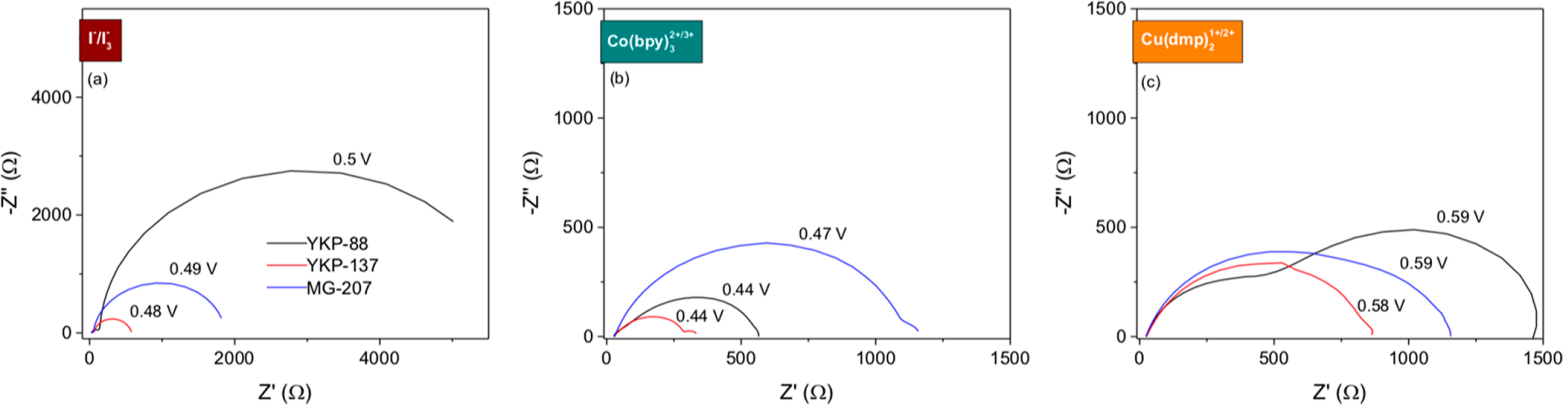
Nyquist plots for the ZnO-based solar cells under blue illumination (λ = 455 nm) at the open-circuit voltage indicated, which is similar to the *V*_OC_ obtained at 1 sun AM1.5G, for the three dyes and redox couples: (a) I^–^/I_3_^–^; (b) Co(bpy)_3_^2+/3+^; (c) Cu(dmp)_2_^1+/2+^.

In order to further analyze the underlying processes that determine the performance of the ZnO-based solar cells and its dependence on the dye and redox couple, a detailed impedance spectroscopy study was performed as a function of the light intensity and temperature. In general, DSSCs may be characterized by up to three arcs,^68,69^ depending on the combination of dye, electrolyte, and light intensity. Figure 5 and Figure S8 in the Supporting Information show a series of Nyquist plots (−imaginary versus real part of the impedance), showing a variety of types of spectra ranging from only one to three semicircles.

For the I^–^/I_3_^–^-based electrolyte solution, two signals that are both independent of temperature (see Figure S9) are observed: a small semicircle at high frequencies (10–50 kHz), which does not change with illumination intensity, followed by a second arc that depends on the light intensity. The high-frequency response corresponds to the impedance related to wires, contacts, and possibly the reduction reaction at the counter electrode. The second and main semicircle observed in the spectra is related to recombination processes taking place within the device,^70^ which is the signal of the most interest in this study. The third commonly observed signal is related to electrolyte diffusion,^71,72^ but this is not observed for the I^–^/I_3_^–^ systems related to the high mobility of the iodide species in the acetonitrile-based electrolyte solution.

The solar cells with the Co(bpy)_3_^2+/3+^ redox couple show three distinct signals; in this case, diffusion of the redox electrolyte is slower and the third signal at lower frequencies can be observed. In Figure S9 it can be observed that this signal depends on temperature with an activation energy of about 30 kJ·mol^–1^, which is in agreement with an ionic diffusion process. These results can be attributed to the smaller diffusion coefficient for the Co(bpy)_3_^2+/3+^ redox couple as reported previously.^61^ For the YKP-88 system, a straight line can be seen between the signal related to the counter electrode and the main recombination arc, which may be attributed to transport processes in the oxide.^70,72^

The devices with the Cu(dmp)_2_^1+/2+^ redox couple produce spectra where only one signal is observed for the YKP-137 and MG-207 dye-sensitized solar cell but two for the YKP-88 dye. The low-frequency signal, in contrast to the observation for the Co(bpy)_3_^2+/3+^ redox couple, does not depend on the temperature but moves to higher frequencies as the light intensity increases until it merges with the first semicircle. Hence, it can be concluded that the low-frequency arc corresponds to the recombination process and not an ionic diffusion process. Surprisingly, the high-frequency loop is found to be dependent on temperature, which implies it corresponds to an activated process with an activation energy of about 54 kJ mol^–1^. These results suggest that the electron transfer process at the counter electrode corresponding to the reduction of Cu(dmp)_2_^2+^ may be slow and can be accelerated by increasing the temperature. In Figure S10 in the Supporting Information, the impedance spectra corresponding to cells with platinum- and PEDOT-catalyzed counter electrodes show a much smaller high-frequency arc for the PEDOT system. These results indicate that indeed PEDOT is a better catalyst for the reduction of Cu(dmp)_2_^2+^, which is in accordance with the interpretation of the better overall performance of the YKP-88 solar cells with PEDOT-catalyzed counter electrode (Figure S7).

The impedance spectra can be fitted to the general DSSC equivalent circuit developed by Bisquert and co-workers.^70,73^ Focusing on the behavior of the main arc related to recombination as a function of the light intensity, the charge transfer or recombination resistance, *R*_CT_, and chemical capacitance, *C*_μ_, can be expressed as a function of the open circuit potential:
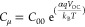
1
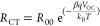
2
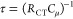
3where *R*_00_ and *C*_00_ are potential-independent constants, α is the trap distribution parameter, β is the recombination coefficient, *q* is the elemental charge, *V*_OC_ is the open-circuit voltage at each light intensity, *k*_B_ is the Boltzmann constant, and *T* is the temperature. The chemical capacitance is defined by the trap state distribution, and if we assume that this is independent of the dye and redox couple but rather determined by the ZnO nanomaterial, the slope of the *C*_μ_ versus *V*_OC_ plot should be a constant, while a shift to higher or lower potentials would indicate a shift of the band edges. Similarly, if the slope of the *R*_CT_ versus *V*_OC_ is a constant, the value of *R*_CT_ at a given *V*_OC_ gives a direct indication of the recombination kinetics. The effective electron lifetime, τ, can be obtained according to eq 3, and both the absolute value and dependence on *V*_OC_ provide information on the contribution of the various factors.

**Figure 6:**
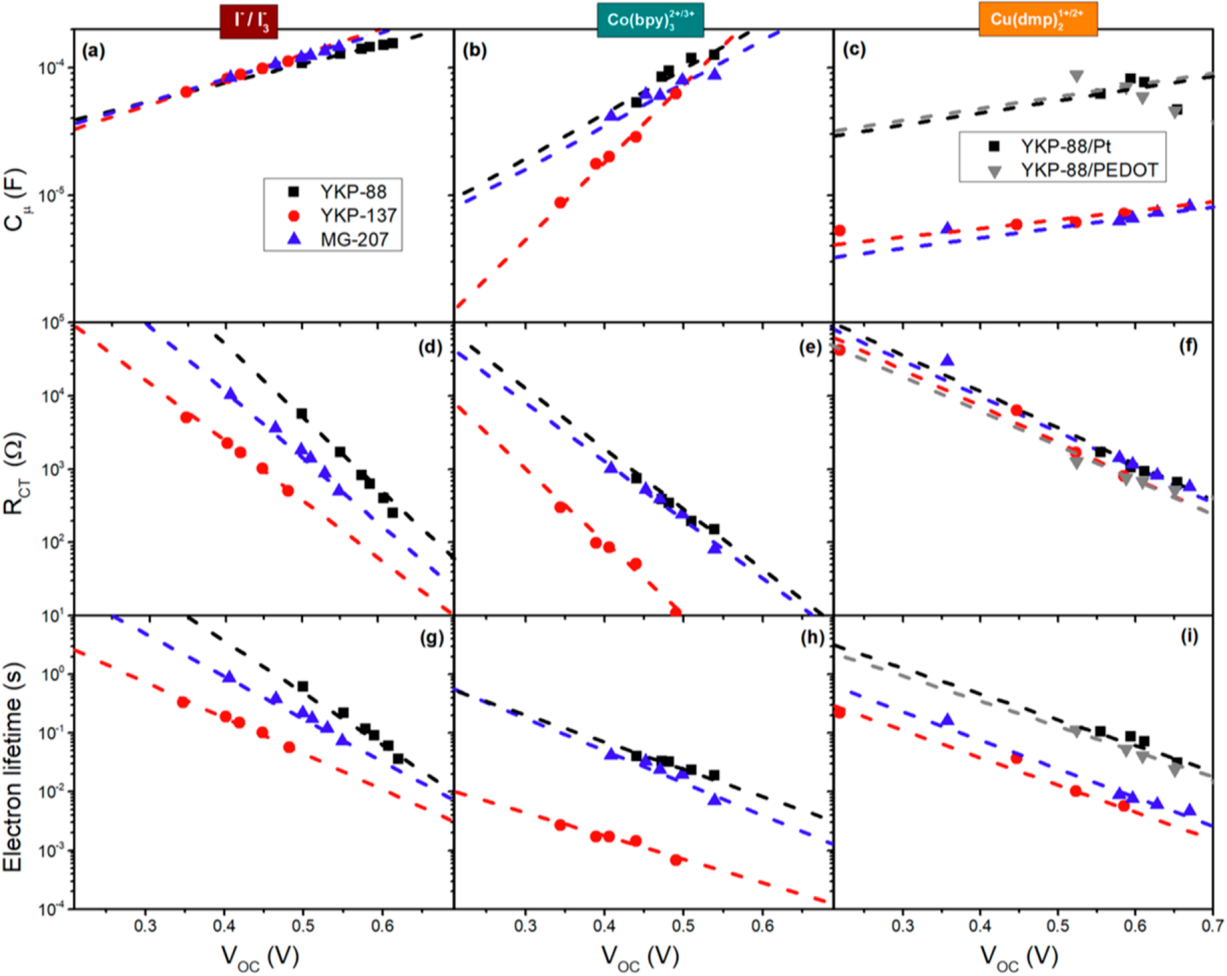
Chemical capacitance (a–c), charge transfer resistance (d–f), and electron lifetime (g–j) as a function of light intensity at the respective open circuit potentials for ZnO-based solar cells using three dyes and three different redox couples (left to right): I^–^/I_3_^–^ (a, d, g); Co(bpy)_3_^2+/3+^ (b, e, h); Cu(dmp)_2_^1+/2+^ (c, f, j). The electrical impedance measurements were performed under monochromatic illumination (λ = 455 nm). The dyes are identified by the color of the data points and trend lines. For the Cu(dmp)_2_^1+/2+^ redox couple (c, f, j), the additional gray square data points correspond to a ZnO/YKP-88 cell with a PEDOT-catalyzed counter electrode.

Figure 6 shows the analysis of the impedance spectra for the nine combinations of dyes and redox couples, providing detailed information on the underlying processes that determine performance. The left column shows the results for the cells with the I^–^/I_3_^–^ redox couple, the middle column corresponds to the cells with Co(bpy)_3_^2+/3+^, while the right column provides the results for the cells with the Cu(dmp)_2_^1+/2+^ redox couple: note that the graphs for the different redox couples have the same values on both *x*- and *y*-axes, making a direct comparison possible.

For the solar cells with the I^–^/I_3_^–^ redox couple, it can be observed that the capacitance increases with light intensity, which is in accordance with eq 1 and an exponential trap state distribution. The results are independent of the dye; hence, no shift of the band edges is evident upon changing the dye chemistry.^60,74^ The recombination resistance exhibits a strong dependence on the dye, which indicates that the recombination rate for electron transfer from ZnO to I_3_^–^ depends on dye chemistry. The cells with YKP-88 have the largest recombination resistance, which results in a larger *V*_OC_ at the same light intensity and, hence, better performance of the cell in agreement with the results shown in Table 1. The recombination resistance decreases somewhat for MG-207 devices and is significantly smaller for the solar cells with YKP-137. These results follow the same tendency as observed in the desorption experiments (Table S3); hence the recombination rate increases at lower dye coverage. This points to an increased ability of the negatively charged I_3_^–^ electron acceptor to approach the ZnO surface highlighting the importance of this aspect.

Upon changing the redox couple in the electrolyte solution, the potential distribution in the solar cell changes, and comparison of the implications of the schematic energy band diagram in Figure 3 with the weak dependence of the *V*_OC_ on redox potential (Table 1) illustrates that this readjustment likely involves band edges shifts and trap distribution changes. The second column shows the impedance analysis for the cells with the Co(bpy)_3_^2+/3+^ redox couple. It can be seen clearly that the slope of the capacitance–voltage plot is steeper than for the I^–^/I_3_^–^ solar cells. This indicates that the trap distribution is different and suggests that the redox couple affects the ZnO surface chemistry. On the other hand, there is a dependence on dye chemistry as well, where the YKP-88 and MG-207 systems show similar behavior and the YKP-137 system exhibits even stronger voltage dependence. Interestingly, at high *V*_OC_, i.e., what would be obtained under 1 sun illumination, the capacitance is similar for all three dyes and essentially equal to that found for the I^–^/I_3_^–^ redox couple.

Comparing the recombination resistance, it can be seen that the slope is not the same but similar for the three dyes and is also similar to that for the I^–^/I_3_^–^ solar cells. The YKP-137 devices show significantly lower recombination resistance than for the YKP-88 and MG-207 devices; in addition, the recombination resistance is generally smaller than for the I^–^/I_3_^–^ solar cells at any *V*_OC_. These results indicate that there is a chemical interaction of the Co(bpy)_3_^2+/3+^ redox couple with the ZnO surface, altering trap state distribution and recombination kinetics, which particularly affects cell performance negatively at low light intensities. The YKP-137 dye performs worse compared to the other two dyes, indicating that the alkoxy side groups allow the positively charged Co(bpy)_3_^2+/3+^ redox to better approach the ZnO surface, resulting in stronger chemical interaction and faster recombination. On the other hand, the much lower dye coverage observed for this system as compared to the other dyes as well as for TiO_2_ ^20^ also directly affects the approachability of the cation and hence the recombination kinetics.

The third column shows the results for the Cu(dmp)_2_^1+/2+^ redox couple, which according to Figure 4 and Table 1 performs significantly less than the other two. Except for the YKP-88 solar cells, the capacitance is significantly smaller than for the cells with the other two redox couples, while the dependence is much less steep than observed for the Co(bpy)_3_^2+/3+^ redox couple and is similar to that of the I^–^/I_3_^–^ solar cells. These observations indicate that a significant realignment of the band edges occurs, specifically for the cells with the YKP-137 and MG-207 dyes, which is in agreement with the observation of smaller *V*_OC_ instead of the thermodynamically expected increase using the Cu(dmp)_2_^1+/2+^ redox couple with a significantly more positive equilibrium potential. It should be noted that the local increase of the concentration of oxidized species of the redox couple due to the regeneration process causes an acceleration of recombination under illumination. The changes in the slopes in the *C*_μ_ and *R*_CT_ versus voltage graphs may be affected by differences in the regeneration rate for the different redox couples. The recombination resistance in this case is independent of dye chemistry. For the YKP-88/Cu(dmp)_2_^1+/2+^ system, the EIS results for both Pt- and PEDOT-catalyzed counter electrodes are shown in the right column of Figure 6. It can be observed that *R*_CT_ and *C*_μ_ are essentially the same for both systems, illustrating that the recombination dynamics are not dependent on the counter electrode catalyst. These results illustrate the usefulness of impedance spectroscopy to obtain quantitative information on processes that occur in different time domains.

The third row of Figure 6 shows the effective electron lifetime for the nine systems obtained from the impedance measurements, and these graphs provide an overall picture of the influence of dye chemistry and redox couple on the solar cell performance. The electron lifetime has been corroborated with intensity-modulated photovoltage spectroscopy (IMVS), which is shown in Figure S11. The electron lifetime for the solar cells with the I^–^/I_3_^–^ redox couple decreases relatively strongly with *V*_OC_, and the recombination resistance mainly defines the dependence on dye chemistry. The trends are similar for the Co(bpy)_3_^2+/3+^ devices; however, the dependence on *V*_OC_ is weaker, and as a consequence, although the electron lifetime is smaller compared to the I^–^/I_3_^–^ redox couple at low light intensities, at 1 sun the electron lifetime is similar or higher, especially for the YKP-88 dye. This explains that the solar cells of the YKP-88/Co(bpy)_3_^2+/3+^ system have better performance at 1 sun and higher light intensities. However, for low light intensity, the I^–^/I_3_^–^ redox couple is shown to work better. On the other hand, the observation of significantly increasing electron lifetime for the three YKP-88 systems at lower light intensity (small *V*_OC_) also makes it possible to observe the transport line in the Nyquist plots,^70,73^ while this is not observed for the other systems.

For the cells with the Cu(dmp)_2_^1+/2+^ redox couple the lifetime is mainly determined by the band edge realignment and the dependence on light intensity is relatively weak. Although the electron lifetime appears to be sufficiently large for good performance for the YKP-88/Cu(dmp)_2_^1+/2+^ system, the band edge energy realignment results in only marginally larger *V*_OC_ and the regeneration kinetics appear to be not optimal to avoid recombination. Note that for this system, the significantly larger electron lifetime for the YKP-88 dye cells as compared to the YKP-137 and MG-207 systems results in the observation of the recombination arc at lower frequency; as a consequence, the Pt-catalyzed counter electrode response is only observed for the YKP-88 dye system. However, as also observed for the capacitance and recombination resistance, the lifetime obtained from the recombination loop does not depend on the counter electrode catalyst, confirming that the analysis of charge dynamics using EIS allows for effective separation of different processes.

Based on this detailed electrochemical analysis, we have identified the YKP-88 dye with both the I^–^/I_3_^–^ and the Co(bpy)_3_^2+/3+^ redox couple for scale-up from small cells to minimodules; in particular, the YKP-88 with Co(bpy)_3_^2+/3+^ system is promising for higher light intensities, i.e., exterior applications, while the YKP-88 with I^–^/I_3_^–^ system is more attractive for lower light intensity systems. It should be noted that the Cu(dmp)_2_^1+/2+^ should not be discarded and may also be a good candidate for up-scaling if optimization of the counter electrode and dye coverage can be achieved, which requires further research.

### Scale-Up to Minimodules

**Figure 7:**
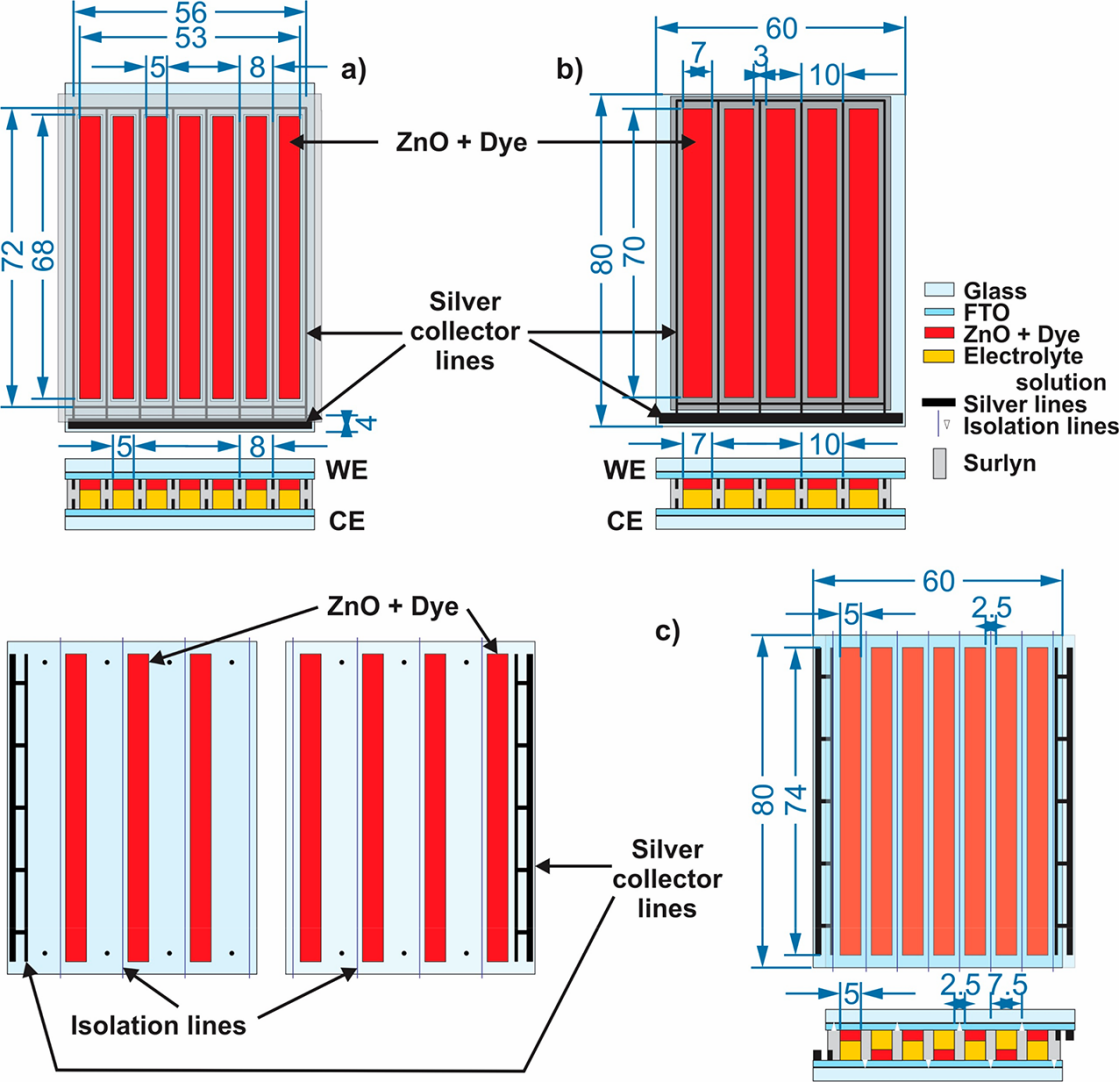
Design and configurations of the minimodules fabricated in this work: (a) 7-strips, parallel connected, active area = 23.8 cm^2^; (b) 5-strips, parallel connected, active area = 24.5 cm^2^; (c) 7-strips, series connected, active area = 25.9 cm^2^. The numbers in blue correspond to the size in mm.

One objective of this work was to demonstrate the fabrication of larger area, semitransparent solar cells with a ZnO photoanode and to investigate different device configurations. To that aim, minimodules were fabricated in the following general steps (for details see the Experimental Section): (i) screen printing of a 10 μm ZnO film onto 6 cm × 8 cm FTO substrates, followed by sintering at 450 °C for 1 h; (ii) screen printing of silver collector lines and curing at 180 °C for 45 min; (iii) immersion in the dye solution for 2 h; (iv) preparation of Pt-catalyzed counter electrode; (v) module assembly using 50 μm Surlyn and a heat press at 230 °C; (vi) injection of the electrolyte solution through holes in counter electrodes; (vii) sealing with Surlyn film and microscope cover glass. The three minimodule designs in several configurations and of an active area larger than 20 cm^2^ are presented in Figure 7.

**Figure 8:**
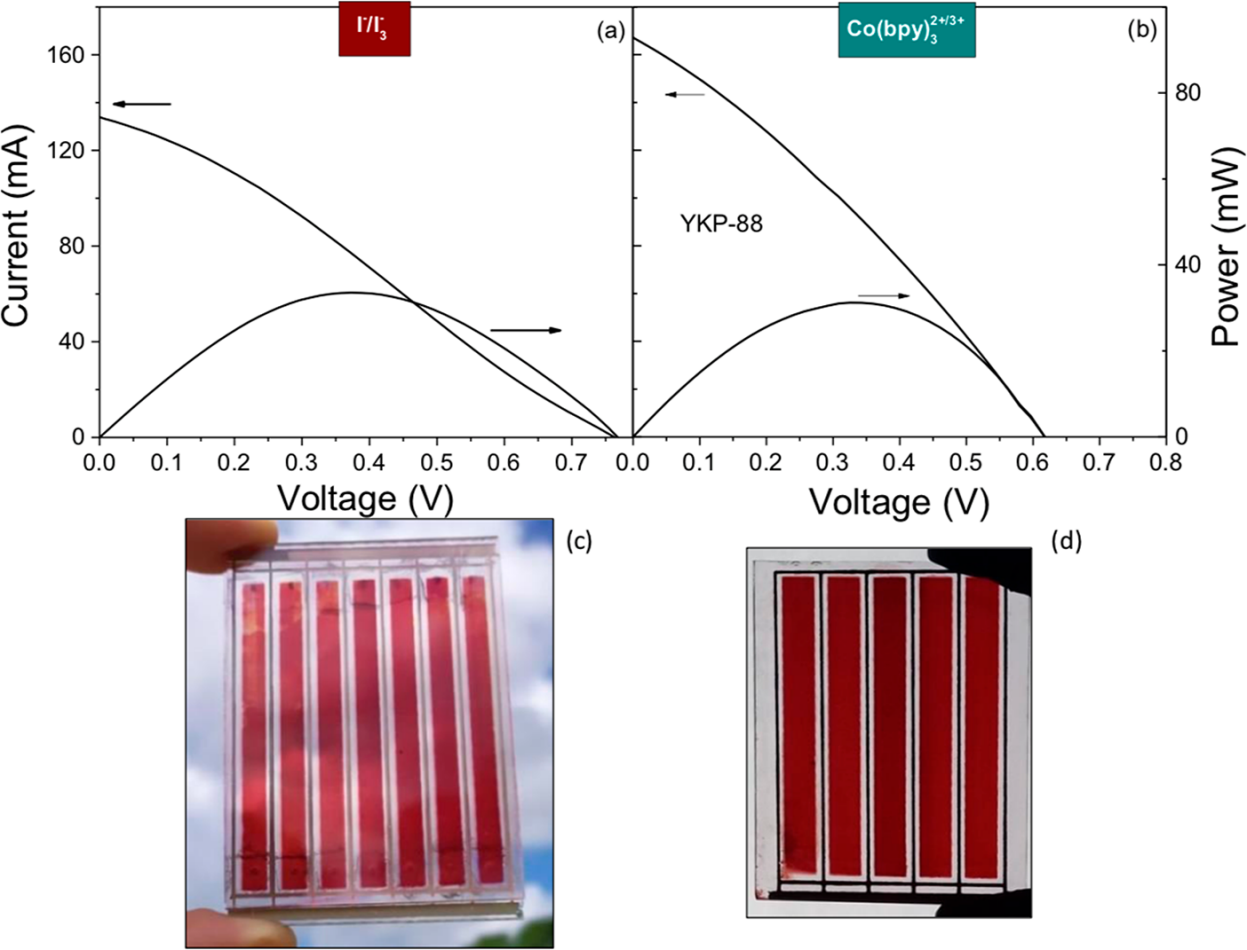
Current–voltage and power–voltage curves for the best ZnO-based minimodules in the parallel configuration under 0.9 sun simulated illumination for (a) 7-strip module with YKP-88 and I^–^/I_3_^–^, with active area of 23.8 cm^2^, and (b) 5-strip module with YKP-88 and Co(bpy)_3_^2+/3+^, with active area of 24.5 cm^2^. Photos of the respective minimodules are shown below the graphs in (c) and (d).

The ZnO-based minimodules with seven strips connected in parallel were sensitized with YKP-88, and the I^–^/I_3_^–^ electrolyte solution was used. This minimodule consists of seven strips of 6.8 cm × 0.5 cm for a total active area of 23.8 cm^2^. The minimodules with five strips connected in parallel were sensitized with YKP-88, and the Co(bpy)_3_^2+/3+^ redox couple was used; this design consists of five strips of 7.0 cm × 0.7 cm for a total active area of 24.5 cm^2^. Note that the total area of both FTO substrates is 48 cm^2^, which includes the area required for contacting the minimodules, illustrating that the (smallest) geometrical fill factor is around 50%; this is an aspect that can be further optimized. Figure 8 shows the current and the total power vs voltage curves under 0.9 sun illumination for the best minimodules for both systems, and Table 2 lists the results.

**Table 2:** Performance Parameters for the Minimodules Reported in Figure 8 under 0.9 Sun (AM1.5G) Illumination^a^

configuration, redox couple	minimodules	*I*_SC_ (mA)	*J*_SC_(mA cm^–2^)	*V*_OC_ (V)	fill factor	PCE (%)
parallel, I^–^/I_3_^–^	average of 3	130.3 ± 13.5	5.4 ± 0.6	0.76 ± 0.01	0.29 ± 0.02	1.3 ± 0.2
parallel, Co(bpy)_3_^2+/3+^	1	166.8	6.8	0.61	0.30	1.4
series, I^–^/I_3_^–^	average of 3	15.4 ± 1.2	4.0 ± 0.4	4.2 ± 0.1	0.42 ± 0.02	1.2 ± 0.2

a Refer to Table S4 in the Supporting Information for the detailed results of each minimodule. The values represent the average for each group and the corresponding standard deviation. The active area is used for the calculation of the PCE and *J*_SC_.

The minimodules in the parallel configuration show a sizable current of up to 166 mA for the module with the Co(bpy)_3_^2+/3+^ redox couple, which corresponds to a photocurrent density of 6.8 mA cm^–2^; this is lower than observed for the small cells (0.5 cm^2^; see Table 1), indicating that current collection is not optimal. This can also be seen from the shape of the curve, which does not exhibit the typical ideal diode shape. This can be attributed to large series resistance as a result of area scaling-up, which is in agreement with the fill factor of 0.3. A shunt resistance may also be present, and the current at 0 V is, in fact, not yet independent of the voltage. The large series resistance indicates that the printed silver lines are not capable of transporting the current without resistive losses. Consequently, the efficiency of the minimodules of up to 1.5% is lower than for small cells; however, we can conclude that within the limitations of a regular laboratory the scale-up has been successful and that with improving the engineering aspect the efficiency can be easily increased.

The minimodules in the parallel configuration with the I^–^/I_3_^–^ redox couple have a lower *J*_SC_, in agreement with the results in Table 1 for the small cells; however, *V*_OC_ is somewhat larger than for the Co(bpy)_3_^2+/3+^ redox couple, thus resulting in about the same overall efficiency. Interestingly, *V*_OC_ is a bit larger than for the small cells for the I^–^/I_3_^–^ redox couples, while it smaller for the minimodule with the Co(bpy)_3_^2+/3+^ redox couple. The latter observation may be a result of the generally less reproducible character of the cells and modules with the Co(bpy)_3_^2+/3+^ redox couple,^21^ particularly for the value of *V*_OC_, which is likely related to the varying level of interaction with the ZnO surface, as concluded from the impedance spectroscopy results.

In order to decrease the impact of series resistance, minimodules were also fabricated in a series configuration, where four strips were printed on one FTO-covered glass substrate of 6 cm × 8 cm and three strips on the other. The cells are isolated by manual scribing and interconnected using printed silver lines. The main advantages of the series connection design are the lower current and higher voltages; in particular, the lower current may avoid the large series resistance losses observed for the parallel configuration.

**Figure 9:**
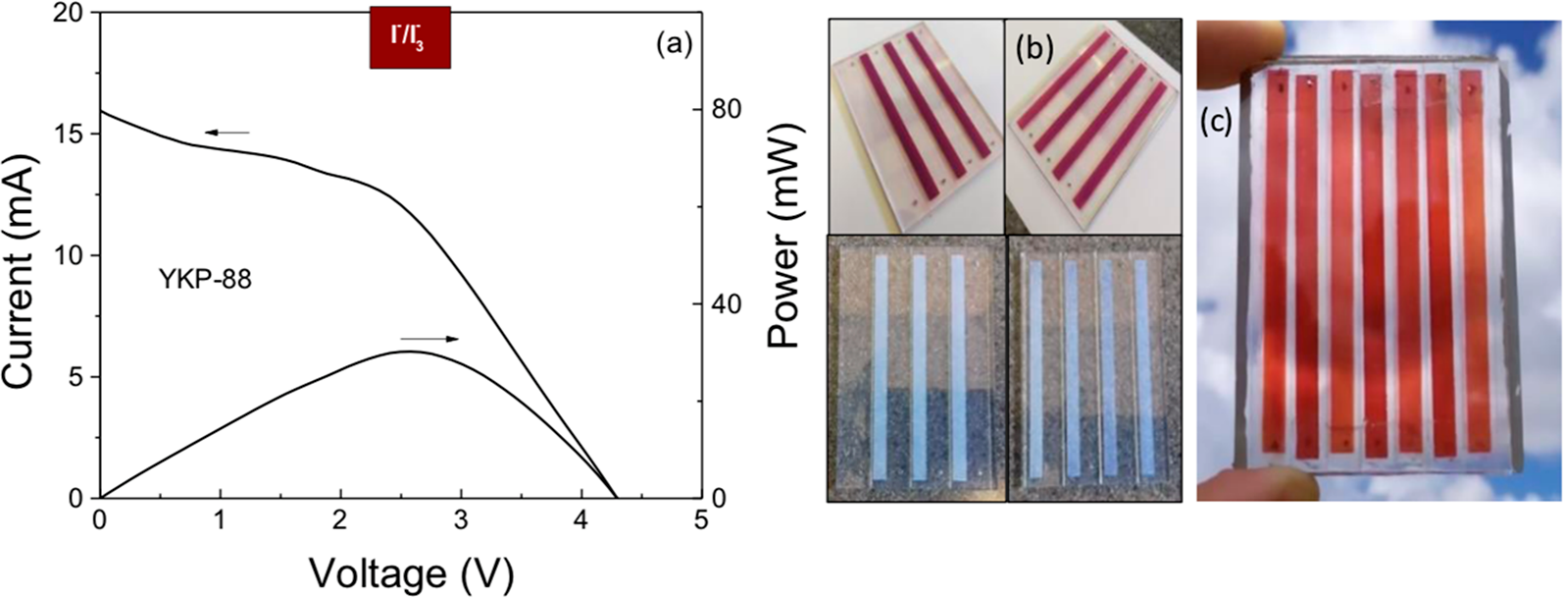
Current–voltage and power–voltage curves for the ZnO-based minimodules in the series configuration under 0.9 sun simulated illumination for 7-strip minimodule with YKP-88 and the I^–^/I_3_^–^ redox couple, with an active area of 25.9 cm^2^. The photos illustrate (b) the design of the minimodule, with 4 strips on one substrate and 3 on the other and (c) the assembled minimodule.

Figure 9 shows the current–voltage curves for three minimodules fabricated according to this design, and the detailed results are shown in Table 2 and Table S4 in the Supporting Information. The best minimodule in the series configuration shows an efficiency of 1.3%, and the *V*_OC_ is about 4.2 V, which is attractive for small-power applications. Although the shape of the curves is much better, with a significant increase in the fill factor, in this case a shunt resistance can also be inferred from the slope of the current–voltage curve at small voltage: this is most likely related to the imprecise manual scribing thus leading to faulty isolation. This problem may be solved with optimized engineering and improved facilities, for example, by using laser scribing.

## Conclusions

We have developed a fast microwave-assisted solvothermal method to synthesize high-quality ZnO nanoparticles for application in dye-sensitized solar cells. The fully organic benzothiadiazole-based dyes YKP-88, YKP-137, and MG-207 with the cyanoacrylic anchoring group, which have previously also shown excellent performance in TiO_2_-based DSSCs, are compared in solar cells and tested using three redox electrolyte solutions with the I^–^/I_3_^–^, Co(bpy)_3_^2+/3+^, and Cu(dmp)_2_^1+/2+^ redox couples, respectively. The best cell performance at 1 sun simulated solar illumination is achieved for the dye–redox couple combination YKP-88 and Co(bpy)_3_^2+/3+^, reaching an average efficiency of 4.7%, compared to 3.7% for the I^–^/I_3_^–^ couple. These values are competitive with record efficiencies reported in the literature with ZnO, specifically for spherical nanoparticle-based ZnO photoelectrodes. The simple synthesis and deposition methods used represent a significant advantage with respect to solar cell with more complex ZnO morphologies, in particular related to scale-up.

Detailed EIS measurements reveal three aspects that complicate the use of the YKP-88/Co(bpy)_3_^2+/3+^ system: (i) the interaction of the redox couple with the ZnO surface prevents a significant increase of the *V*_OC_ with respect to that of the I^–^/I_3_^–^ system; (ii) at lower light intensity, the electron lifetime decreases more strongly than for the I^–^/I_3_^–^ system, indicating that the higher efficiency obtained at 1 sun does not translate to better performance at lower light intensities. As a consequence, the system with the Co(bpy)_3_^2+/3+^ redox couple is expected to be more sensitive to fluctuations in light intensity under real, outdoor circumstances; and (iii) at low frequency, a thermally activated transport process is observed, indicating that for larger current densities, the relatively small Co(bpy)_3_^2+/3+^ diffusion coefficients may affect performance. The Cu(dmp)_2_^1+/2+^ redox system does not work well for any of the dyes, which appears to be related to slow regeneration kinetics resulting in lower *J*_SC_ but also interaction with ZnO leading to relatively small *V*_OC_. The recombination kinetics for the Cu(dmp)_2_^1+/2+^ redox system are relatively fast, resulting in overall unsatisfactory performance for the ZnO/organic dye systems studied in this work. In addition, the counter electrode kinetics are found to significantly affect the performance: when using the usual Pt catalyst, a thermally activated process is observed that corresponds to reduction of Cu(dmp)_2_^2+^ at the counter electrode. This can be avoided by implementing a PEDOT-catalyzed counter electrode, where this effect is not observed.

On the other hand, the dye chemistry mainly affects the recombination kinetics, through interactions with the redox couples, also depending on adsorbed dye density. The YKP-88 dye combines the best properties in both aspects, while the YKP-137 has the worst performance. In particular, the electron lifetime is significantly smaller in the case of the Co(bpy)_3_^2+/3+^ redox system, implying that transport of the positively charge redox species to the ZnO surface is not inhibited by the additional alkoxy side groups that define YKP-137. The YKP-137 dye has a lower adsorption density than the other two dyes, which also affects the recombination kinetics negatively. On top of that, in the case of the YKP-137/Cu(dmp)_2_^1+/2+^ system, the regeneration kinetics limit the performance significantly.

Based on the photovoltaic performance of the small cells, we have fabricated minimodules for two dye/redox couple systems and in two module configurations: parallel and in series. In both configurations, the efficiencies are limited due to engineering issues and manual manufacture of the minimodules, but a clear proof of concept is present. In all systems, the YKP-88 dye performed best, but both the Co(bpy)_3_^2+/3+^ and I^–^/I_3_^–^ redox systems are shown to be viable. A minimodule of the YKP-88/Co(bpy)_3_^2+/3+^ system in the parallel configuration with total active area of 24.8 cm^2^ achieved a 1.4% efficiency, with a short circuit current of 0.17 A and a *V*_OC_ of 0.61 V. On the other hand, the best minimodule of the YKP-88/I^–^/I_3_^–^ system in the parallel configuration with total active area of 23.8 cm^2^ achieved a 1.5% efficiency, with a somewhat lower short circuit current of 0.14 A but higher *V*_OC_ of 0.76 V. The fill factors for all parallel devices are found to be low at about 0.30, being an important reason for the lower efficiencies as compared to the small cells. The series configuration significantly improves the fill factor, and a minimodule of the YKP-88/I^–^/I_3_^–^ system with total active area of 25.9 cm^2^ achieved an efficiency of 1.3%, with a short-circuit current of 15.9 mA, an open circuit voltage of 4.28 V, and a fill factor of 0.44. In this case, the performance appears to limit to the presence of a shunt resistance, related to the manually applied insulation lines.

It can be concluded that detailed analysis of the mechanisms limiting the performance of ZnO-based DSSCs using three fully organic dyes and three different redox couples provides important fundamental insights and useful guidelines for the development ZnO-based DSSCs by highlighting ZnO/dye/electrolyte solution interplay. This study paves the way for new studies and strategies for chemical engineering of organic dyes and coordination complex-based redox couples to improve the efficiency of ZnO-based solar systems.

## Experimental Section

### Chemicals and Instruments

Most chemicals were purchased from Sigma-Aldrich at ACS Reagent grade and used as received: zinc acetate dihydrate (≥98%); chenodeoxycholic acid (CDCA) (≥97%); isopropyl alcohol (IPA); ethyl cellulose (viscosity 100 cP); terpineol (mixture of isomers; anhydrous); lithium iodide (99.9%); iodine (99.8%); guanidine thiocyanate (≥97%); acetonitrile (99.93%); lithium perchlorate (≥95%); bis(trifluoromethane)sulfonimide lithium salt (99.95%); chloroform (≥99.5%); 4-*tert*-butylpyridine (96%); sodium dodecyl sulfate (≥98.5%); 3,4-ethylenedioxythiophene (97%). The redox couple salt Co(2,2′-bpy)_3_[B(CN)_4_]_2_ was purchased from Eversolar, and Co(2,2′-bpy)_3_[B(CN)_4_]_3_, Cu(dmp)_2_TSFI, and Cu(dmp)_2_TFSI Cl were from Dyenamo. The organic dyes YKP-88, YKP-137, and MG-207 were synthesized by the Demadrille group; see ref (65) for experimental details on the synthesis. Absolute ethyl alcohol (ACS Reagent grade) was bought from Macron. The platinum deposition solution (Platisol T) and 1-butyl-3-methylimidazolium iodide (BMII) were purchased from Solaronix.

A rotary evaporator (BUCHI Rotavapor R-210) was used for the ZnO paste preparation. The ZnO films were deposited using a semiautomatic screen printer (ATMA: AT-25PA digital electric flat screen printed). The thickness of ZnO films was measured by profilometry (KLA-Tencor AlphaStep D-120). The ZnO film morphology was visualized using a JEOL JSM-7600F field emission scanning electron microscope. The crystallographic aspects were determined with a Siemens D5000 X-ray powder diffractometer. Photovoltaic characterization of small cells (0.5 cm^2^) was performed using a setup consisting of a 450 W ozone-free Xe lamp (Oriel) with a water filter and an AM 1.5G optical filter. The intensity was calibrated using a certified 4 cm^2^ monocrystalline silicon reference cell with a KG-5 filter incorporated. For the *J*–*V* measurement, a black mask (1 cm × 0.5 cm) was used to avoid additional contributions of scattered light in the glass. The spectra were recorded with a potentiostat AutoLab PGSTAT302N setup, and NOVA 2.1 software was used for data analysis. The photovoltaic performance of the minimodules was determined using an Oriel Sol2A class ABA solar simulator calibrated using a certified reference cell.

### Synthesis of ZnO and Preparation of Screen Printing Paste

The ZnO nanoparticles were synthesized using a microwave-assisted solvothermal method using a MARS 6 (CEM Analytical): 4.39 g of zinc acetate dihydrate was added to 50 mL of pure ethyl alcohol followed by the addition of 0.72 mL of deionized water. The resultant solution was microwave treated at 150 °C for 20 min and at 400 W. The final suspension was decanted, and the ZnO was washed and dried. On the other hand, the screen-printing paste was prepared by dispersing 0.5 g of ZnO nanomaterial in 10 mL of ethanol, followed by sonication for 30 min. Simultaneously, 0.15 g of ethyl cellulose was dissolved in 10 mL of ethanol for 2 h using an ultrasonic bath; 4.06 g of terpineol was added to the ZnO dispersion under sonication for 30 min. Then, both solutions were mixed and sonicated for another 30 min. Finally, the mixture was condensed by removing the excess ethanol until the paste was viscous enough to be used for screen-printing.

### Organic Dyes and Electrolyte Solutions

The organic dyes evaluated in this work are labeled YKP-88, YKP-137, and MG-207. Sensitization was performed from 0.2 mM dye + 2 mM CDCA dissolved in a mixture of CHCl_3_ and ethanol (1:1 v/v). The amount of dye adsorbed was determined by desorbing the dye in a known volume of 0.1 M KOH in methanol; UV–vis spectrophotometry was performed to determine the absorbance at the wavelength of the maximum, and using the absorption coefficient reported previously, the amount of dye adsorbed was obtained.^65^

The HOMO energy levels of the three dyes in acetonitrile-based electrolyte solutions were obtained from cyclic voltammetry for YKP-88, YKP-137, and MG-207 adsorbed onto ZnO films in 0.1 M TBAPF_6_ in acetonitrile at a scan rate of 50 mV s^–1^ and at 25 °C. The reference electrode consisted of a silver wire in 0.01 M AgNO_3_ in acetonitrile, and the counter electrode was a Pt wire. The dyed ZnO films were prepared using a sensitization time of 2 h in 0.2 mM organic dye + 2 mM CDCA in CHCl_3_/ethanol (1:1 v/v).

The redox electrolyte solution based on the I^–^/I_3_^–^ redox couple consisted of 1.02 M BMII, 0.03 M I_2_, 0.1 M GuSCN, 0.52 M TBP, and 0.05 M LiI in acetonitrile. The solution with the Co(bpy)_3_^2+/3+^ redox couple was prepared using 0.22 M Co(bpy)_3_ [(B(CN)_4_)_2_], 0.05 M Co(bpy)_3_ [(B(CN)_4_)_3_], 0.1 M LiClO_4_, and 0.42 M TBP in acetonitrile. Finally, the third electrolyte solution based on the Cu(dmp)_2_^1+/2+^ redox couple was prepared using 0.2 M Cu^I^(dmp)_2_TFSI, 0.05 M Cu^II^(dmp)_2_TFSI Cl, 0.1 M LiTFSI, and 0.5 M TBP in acetonitrile.

The redox potentials were determined using cyclic voltammetry at a scan rate of 50 mV/s in diluted acetonitrile-based solutions of the reference redox couple ferrocene/ferrocenium and the three redox couples mimicking the general electrolyte chemistry of the solar cell solutions, using Pt wire as working and counter electrodes and Ag/0.01 M AgNO_3_ in acetonitrile as reference electrode. The redox potentials were taken at the average half-wave potential. Ferrocene^0/+^: 2 mM ferrocene + 0.1 M TBAPF_6_. I^–^/I_3_^–^: 7.5 mM BMII + 0.22 mM I_2_ + 0.73 mM GuSCN + 3.8 mM TBP + 0.36 mM LiI + 0.1 M TBAPF_6_. Co(bpy)_3_^2+/3+^: 2.75 mM Co(bpy)_3_[(B(CN)_4_)_2_] + 0.625 mM Co(bpy)_3_[(B(CN)_4_)_3_] + 1.25 mM LiClO_4_ + 2.5 mM TBP + 0.1 M TBAPF_6_. Cu(dmp)_2_^1+/2+^: 10 mM Cu^I^(dmp)_2_TFSI + 2.5 mM Cu^II^(dmp)_2_TFSI Cl + 5 mM LiTFSI + 25 mM TBP + 0.1 M TBAPF_6_.

### Solar Cell Assembly

Dye-sensitized solar cells with an active area of 0.5 cm^2^ were fabricated with ZnO photoanodes deposited by screen-printing onto FTO conductive glass substrates (TEC-15), which were previously cleaned with soap, deionized water, ethanol, and isopropanol in an ultrasonic bath. The ZnO films were sintered at 450 °C for 1 h using a controlled ramp program (Figure S12 in the Supporting Information). The ZnO film thickness was determined by profilometry at 11 μm. After cooling down to 100 °C, the electrodes were immersed in a dye solution for two hours. The counter electrodes were prepared by spreading a drop of Platisol T on the FTO conductive glass substrates (TEC-8) and subsequently heated at 400 °C for 5 min. PEDOT counter electrodes were prepared according to the procedure previously published.^67^ An aqueous solution of 0.1 M sodium dodecyl sulfate (SDS) and 0.01 M 3,4-ethylenedioxythiophene (EDOT) was used as a working solution. Both solutions were sonicated separately for 1 h in order to achieve complete dissolution. Electropolymerization of EDOT was performed using a chronopotentiometric method with a potentiostat/galvanostat/ZRA GAMRY reference 3000 in a two-electrode cell configuration with a platinum mesh as counter electrode and a FTO on glass substrate (TEC 7; 2 cm × 2 cm) as working electrode. A constant current of 2 mA was applied for 25 s. After deposition, the electrodes were rinsed with water and ethanol and dried in air.

The photoanode and counter electrode were assembled into a sandwich-type solar cell configuration using a frame of the thermoplastic Surlyn and were sealed at 200 °C for 2.5 min. Finally, the electrolyte solution was introduced into the cells through previously perforated holes in the counter electrode, which were subsequently sealed with Surlyn and microscope cover glass.

### Minimodule Assembly

The minimodules were fabricated on 6 cm × 8 cm FTO substrates, using TEC 15 and TEC 8 y as the working and counter electrodes, respectively. The substrates were cleaned using the same procedure as for small cells. ZnO was deposited using screen printing, and the films were sintered at 450 °C for 1 h. The silver collector lines were also deposited by screen printing using commercial Ag paste (Dyesol) between the strips of ZnO and were cured at 180 °C for 45 min. The substrates were immersed in the dye solution for 2 h. The ZnO film thickness was 10 μm for all minimodules. The counter electrodes were prepared by drilling two holes for each strip, and the platinum solution was deposited via drop casting followed by heat treatment at 400 °C for 5 min. The minimodules were assembled with 50 μm Surlyn and using a T-shirt heat press at 230 °C. The electrolyte solution was injected through the holes in counter electrodes and sealed with a Surlyn and cover glass.

### Optoelectronic Characterization

All small-signal perturbation characterization techniques (EIS and IMVS) were done using an Autolab/PGSTAT302N potentiostat, coupled with a FRA32 module through an LED communication driver (Autolab). EIS measurements were conducted by applying the open-circuit voltage obtained under constant illumination using a blue (455 nm) LED over a wide range of light intensities. A 10 mV perturbation in the 10^–1^–10^5^ Hz frequency domain was applied. NOVA 2.1 software was used to communicate with the potentiostat and LED Driver, and Z-View equivalent circuit modeling software (Scribner) was used to fit the spectra according to the equivalent circuit model developed by Bisquert and co-workers for DSSCs.^70,73^ IMVS measurements were performed in a frequency range restricted to 10^–1^–10^4^ Hz, related to technical limitations of the LED-based setup. The perturbation amplitude of the light illumination was set to 10% of the DC background illumination intensity, calibrated with an FDS100 Thorlabs silicon photodiode.

An additional EIS study was performed controlling the temperature in a range of 278–318 K using a chamber isolated from the external environment. A flux of nitrogen was used to decrease the temperature, while an electrical resistor allowed for increasing the temperature of the chamber. The light from the external LED reached the solar cell through a quartz window included in the chamber. The resulting temperature dependence of the time constants was used to determine the activation energy of the process applying Arrhenius’ law:
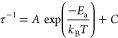
4where τ is the temperature-dependent time constant, *A* is the Arrhenius prefactor, *E*_a_ is the activation energy, and *C* is a constant.

## Supporting Information Available

X-ray diffraction pattern of ZnO nanomaterial; cyclic voltammetry of the three dyes on ZnO; cyclic voltammetry and steady-state current potential curves of the three redox couples; quantitative data for the energies and potentials of the dyes and redox couples; UV–vis spectra for dye desorption measurements and dye coverage data; *J*–*V* curves of solar cells organized by redox couple; temperature dependence of *V*_OC_ of solar cells; additional *J*–*V* curves and Nyquist plots for the YKP-88/Cu(dmp)_2_ system with Pt and PEDOT-catalyzed counter electrode; additional Nyquist plots from the EIS measurements for the nine systems; temperature dependence of time constants derived from EIS; additional results from intensity-modulated photovoltage spectroscopy (IMVS); quantitative data from the minimodule characterization; electrode heat treatment program (PDF)
