# Supplementary material for: Influence of Redox Couple on the Performance of ZnO Dye Solar Cells and Minimodules with Benzothiadiazole-Based Photosensitizers
Source: ACS Appl Energy Mater. 2022 Nov 8;5(11):14092–106. doi: 10.1021/acsaem.2c02609 (PMC9709824; doi:10.1021/acsaem.2c02609)
Supplement: Supplementary file 1 — ae2c02609_si_001.pdf [file ae2c02609_si_001.pdf]

# Supporting Information

## Influence of redox couple on the performance of ZnO dye solar cells and mini-modules with benzothiadiazole-based photosensitizers

Carlos A. Gonzalez-Flores*, Dena Pourjafari, Renan Escalante, Esdras J. Canto-Aguilar, Alberto Vega Poot, José Maria Andres Castán, Yann Kervella, Renaud Demadrille, Antonio J. Riquelme, Juan A. Anta and Gerko Oskam*

[a] *Departamento de Física Aplicada, CINVESTAV-IPN, Antigua Carretera a Progreso km 6, Mérida 97310, Yucatán, México.*

[b] *Área de Química Física, Departamento de Sistemas Físicos, Químicos y Naturales, Universidad Pablo de Olavide, ES-41013, Seville, Spain.*

[c] *Facultad de Ingeniería, Universidad Autónoma de Campeche-Campus V, San Francisco de Campeche, Campeche 24085, México.*

[d] *Université Grenoble Alpes, CEA, CNRS, IRIG-SyMMES, Grenoble 38000, France.*

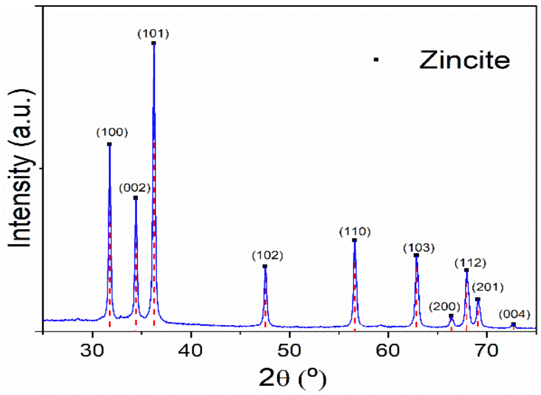


**Figure S1.** X-ray diffraction pattern of the ZnO nanomaterial in powder form prepared by the microwave-assisted solvothermal method.

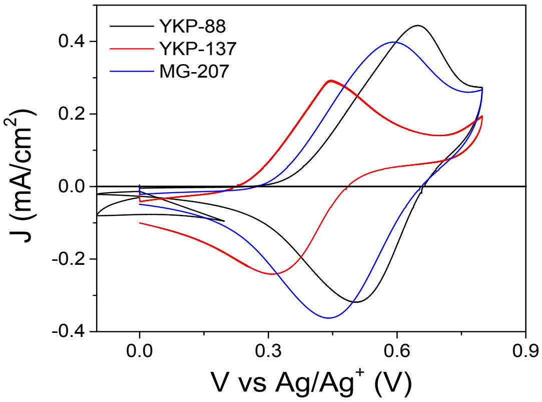


**Figure S2.** Cyclic voltammetry for YKP-88, YKP-137 and MG-207 adsorbed onto ZnO films in 0.1 M $TBAPF_6$ in acetonitrile at a scan rate of 50 mV $s^{-1}$ and at 25°. The reference electrode consisted of a silver wire in 0.01 M $AgNO_3$ in acetonitrile, and the counter electrode was a Pt wire. The dyed ZnO films were prepared using a sensitization time of 2 hours in 0.2 mM organic dye + 2 mM CDCA in $CHCl_3$/ethanol (1:1 v/v).

**Table S1.** Data obtained from the cyclic voltammetry results in Figure S2 for the sensitized ZnO films in 0.1 M $TBAPF_6$ in acetonitrile.

| | YKP-88 / ZnO | YKP-137 / ZnO | MG-207 / ZnO |
|---|---|---|---|
| $V_{ox}$ vs $Ag/Ag^+$ (V) | 0.65 | 0.45 | 0.59 |
| $V_{red}$ vs $Ag/Ag^+$ (V) | 0.49 | 0.31 | 0.44 |
| $V_{HOMO}$ vs $Ag/Ag^+$ (V) | 0.57 | 0.38 | 0.52 |
| $V_{HOMO}$ vs NHE (V) | 1.11 | 0.92 | 1.06 |
| $V_{LUMO}$ vs NHE (V) | -0.89 | -0.90 | -0.89 |
| $E_{HOMO}$ * (eV) | -5.28 | -5.09 | -5.23 |
| $\lvert E_{HOMO-LUMO}\rvert$ ** (eV) | 2.00 | 1.82 | 1.95 |
| $E_{LUMO}$ (eV) | -3.28 | -3.27 | -3.28 |

* The HOMO energies were calculated using $E^o(Fc^{0/+})$ = -4.80 eV vs vacuum,[1] with the reference electrode calibrated according to Table S2 resulting in $E(Ag/Ag^+)$ = -4.89 eV.
** The values for $\lvert E_{HOMO-LUMO}\rvert$ were taken from our previous work, where electrochemistry in dichloromethane was used to determine both the HOMO and LUMO energies of the three dyes; note that the values are essentially identical to the optical gap determined experimentally.[1]

**Table S2.** Redox potentials of the electrolyte solutions determined by electrochemical measurements as detailed in the caption of Figure S3, and in accordance with previous work.[1-3]

| Redox couple | V° vs $Ag/Ag^+$ (V) | V° vs NHE (V) |
|---|---|---|
| $Fc^{0/+}$ | 0.09 | 0.63 |
| $I^-/I_3^-$ | -0.18 | 0.36 |
| $Co(bpy)_3^{2+/3+}$ | 0.01 | 0.55 |
| $Cu(dmp)_2^{1+/2+}$ | 0.37 | 0.91 |

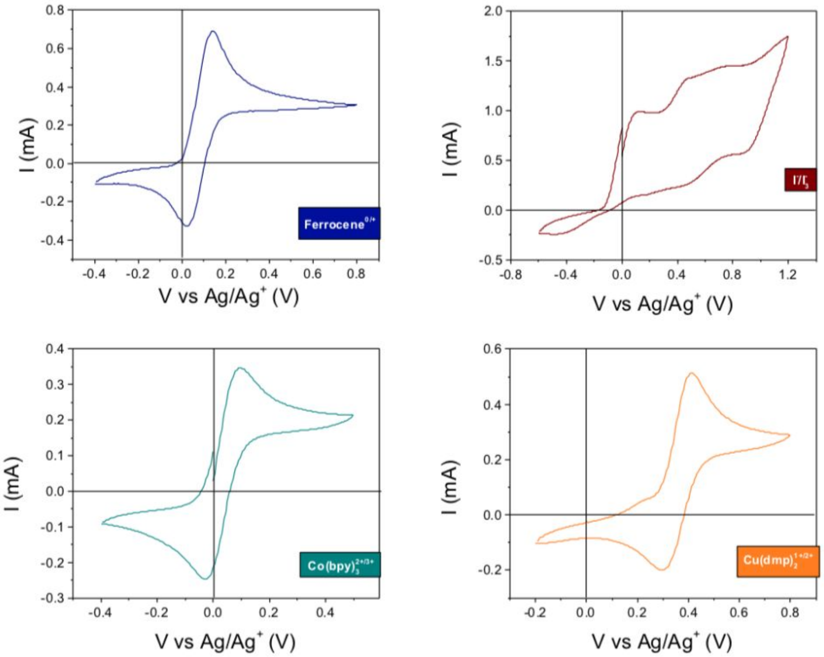


**Figure S3.** Cyclic voltammetry at a scan rate of 50 mV/s in diluted acetonitrile-based solutions of the reference redox couple ferrocene / ferrocenium and the three redox couples mimicking the general electrolyte chemistry of the solar cell solutions, using Pt wire as working and counter electrodes, and Ag / 0.01 M $AgNO_3$ in acetonitrile as reference electrode. The redox potentials were taken at the average half-wave potential. **Ferrocene$^{0/+}$**: 2 mM ferrocene + 0.1 M $TBAPF_6$; **$I^-/I_3^-$**: 7.5 mM BMII + 0.22 mM $I_2$ + 0.73 mM GuSCN + 3.8 mM TBP + 0.36 mM LiI + 0.1 M $TBAPF_6$ ; **$Co(bpy)_3$ $^{2+/3+}$**: 2.75 mM $Co(bpy)_3$ $[(B(CN)_4)_2]$ + 0.625 mM $Co(bpy)_3$ $[(B(CN)_4)_3]$ + 1.25 mM $LiClO_4$ + 2.5 mM TBP + 0.1 M $TBAPF_6$ ; **$Cu(dmp)_2$ $^{1+/2+}$**: 10 mM $Cu^I(dmp)_2$ TFSI + 2.5 mM $Cu^{II}(dmp)_2$ TFSI Cl + 5 mM LiTFSI + 25 mM TBP + 0.1 M $TBAPF_6$.

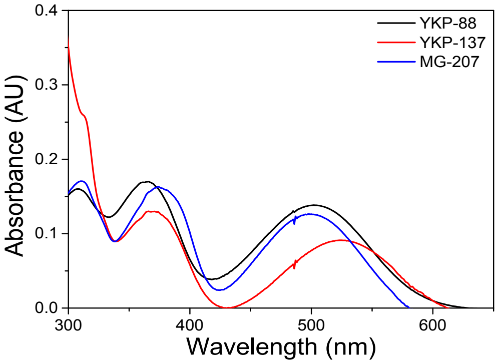


**Table S3:** Adsorbed dye density on ZnO expressed per $cm^2$ of geometric area.

| | YKP-88 | MG-207 | YKP-137 |
|---|---|---|---|
| Adsorbed dye ($10^{-8}$ mol $cm^{-2}$) | **7.8** | **7.2** | **5.3** |

**Figure S4.** UV-Vis spectra of the desorbed dyes using a 0.1 M KOH solution in methanol. Using the absorption coefficients at the maximum in the visible region from previous work,[1] the amount of dye was obtained and is given in Table S3.

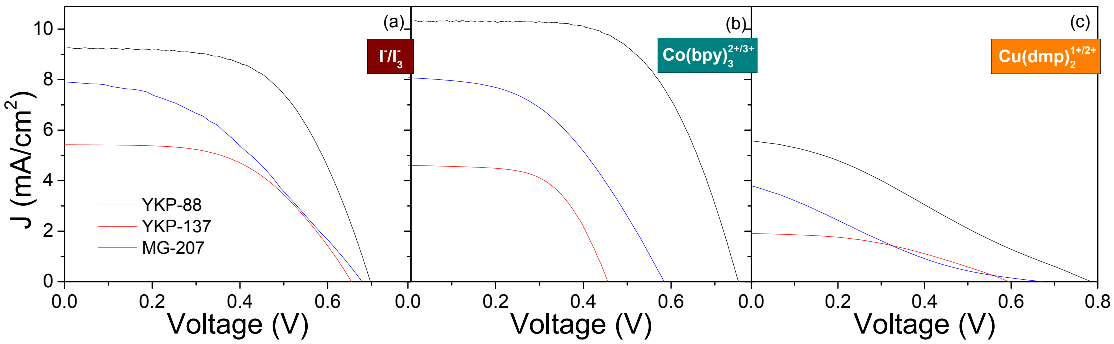


**Figure S5.** *J-V* curves under 1 sun (AM1.5G) illumination of 0.5 cm$^2$ ZnO-based solar cells fabricated with the three different redox electrolyte solutions as a function of dye chemistry.

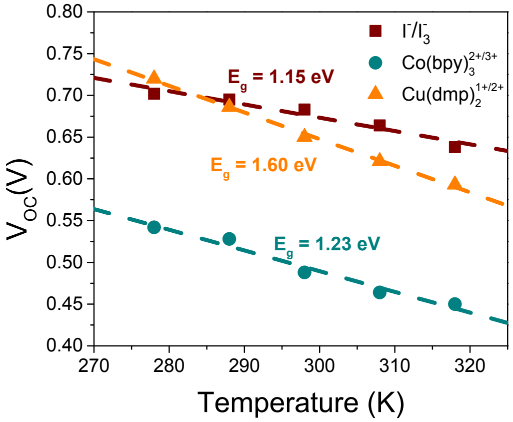


**Figure S6.** Temperature dependence of the $V_{OC}$ for YKP-88 dye-sensitized ZnO solar cells with the three different redox electrolytes.

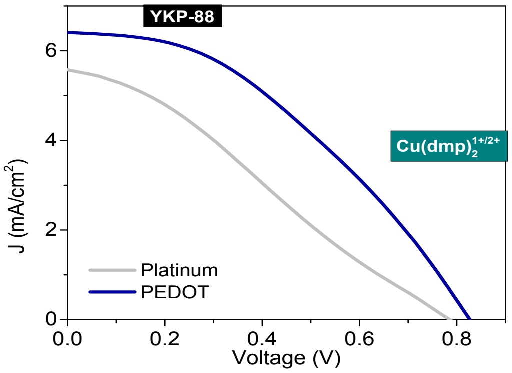


**Figure S7.** *J-V* curves under 1 sun illumination of ZnO cells fabricated with YKP-88 and the Cu(dmp)$_2{}^{1+/2+}$ redox couple for two different counter electrode catalysts: Pt and PEDOT.

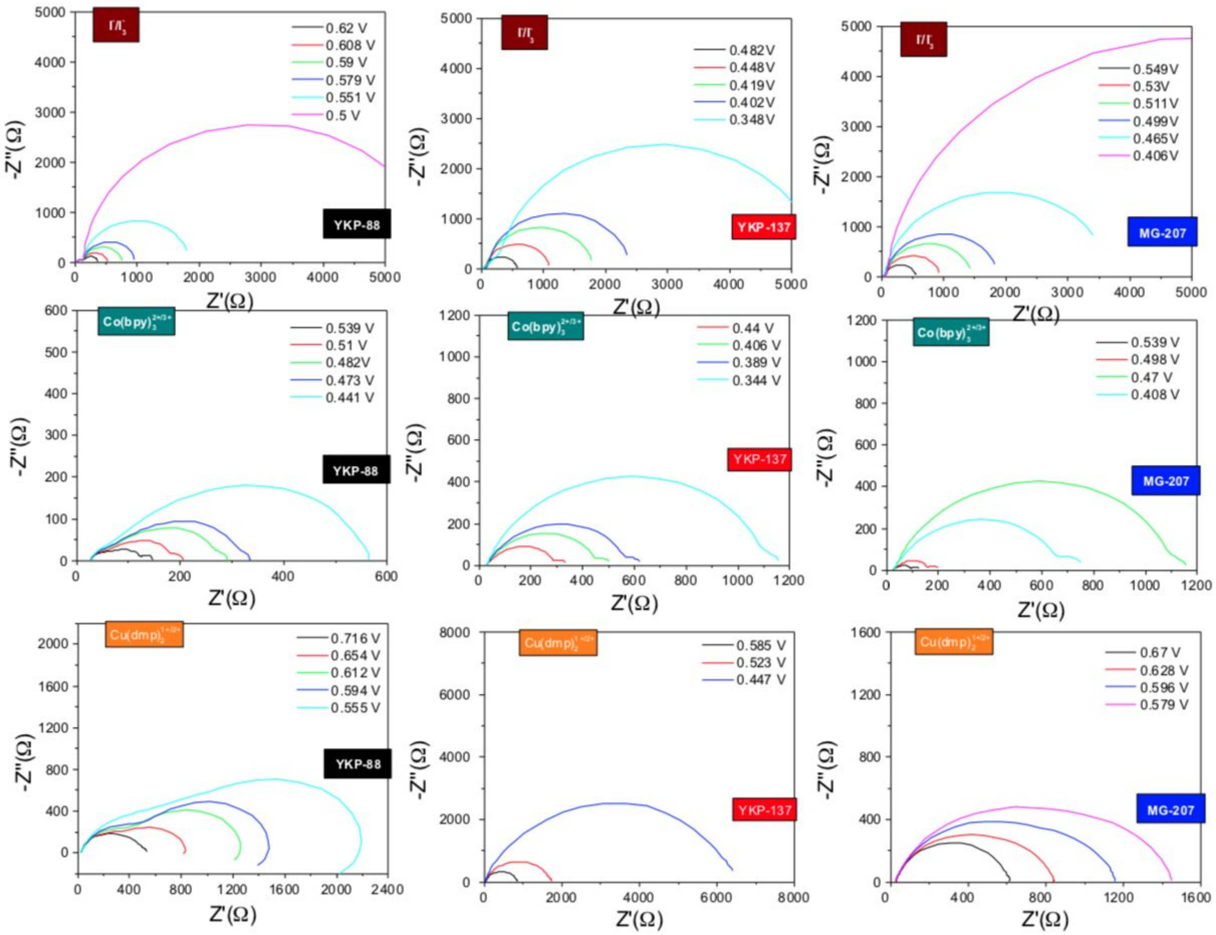


**Figure S8.** EIS results under blue illumination and applying the $V_{OC}$ obtained at different light intensities, for a YKP-88 (left), YKP-137 (middle) and MG-207 (right) dye-sensitized solar cell using $I^-/I_3^-$ (top), $Co(bpy)_3{}^{2+/3+}$ (middle), and $Cu(dmp)_2{}^{1+/2+}$ (bottom) as redox couples.

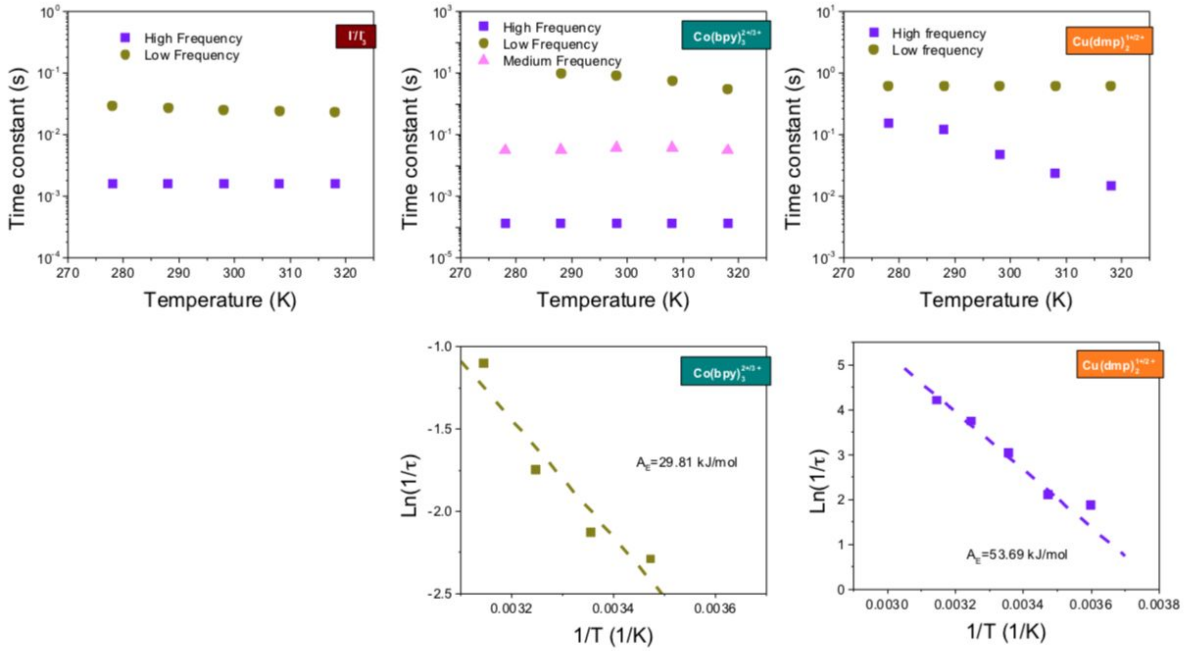


**Figure S9.** Temperature dependence of the time constants observed in EIS measurements of YKP-88 dye-sensitized solar cells using $I^-/I_3^-$, $Co(bpy)_3$ $^{2+/3+}$, and $Cu(dmp)_2$ $^{1+/2+}$ electrolyte solutions. Below, modified Arrhenius plots with the signals that showed a temperature dependence: the low frequency signal for the $Co(bpy)_3$ $^{2+/3+}$ solution; and the high frequency signal for the $Cu(dmp)_2$ $^{1+/2+}$ solution. The respective activation energies are indicated.

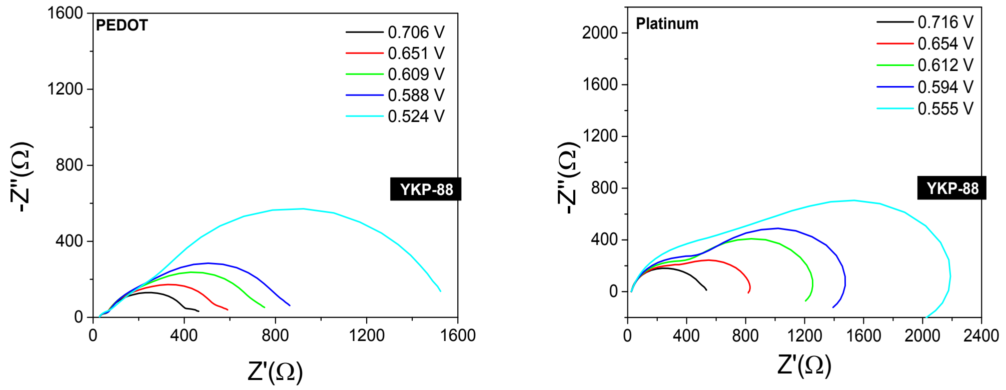


**Figure S10.** Impedance measurements using platinum and PEDOT as a counter electrode catalysts for the ZnO cells fabricated with YKP-88 and the $Cu(dmp)_2$$^{1+/2+}$ redox couple.

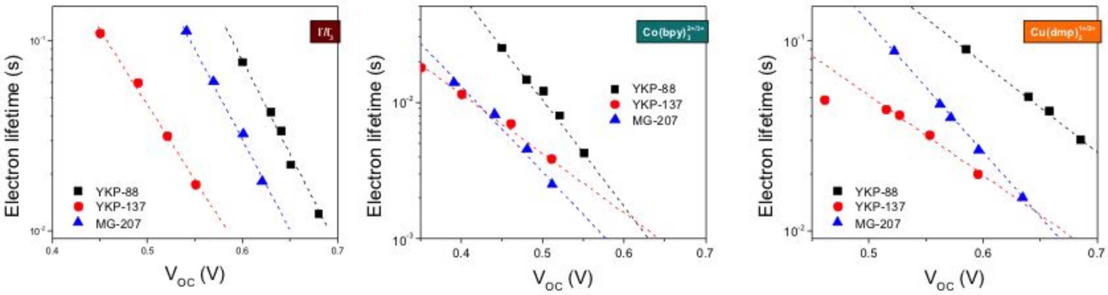


**Figure S11.** Recombination lifetime extracted from IMVS measurements.

**Table S4.** Results for the mini-modules fabricated in this work.

| Configuration; Redox couple | module | $I_{SC}$ (mA) | $J_{SC}$ (mA cm$^{-2}$) | $V_{OC}$ (V) | *Fill factor* | *PCE* (%) |
|---|---|---|---|---|---|---|
| Parallel; $I^-/I_3^-$ | 1 | 133.3 | 5.6 | 0.76 | 0.28 | 1.3 |
| | 2 | 115.5 | 4.8 | 0.77 | 0.29 | 1.2 |
| | 3 | 142.1 | 5.9 | 0.76 | 0.31 | 1.5 |
| | **average** | **130.3** | **5.4** | **0.76** | **0.29** | **1.3** |
| Parallel; $Co(bpy)_3^{2+/3+}$ | **1** | **166.8** | **6.8** | **0.61** | **0.30** | **1.4** |
| Series; $I^-/I_3^-$ | 1 | 15.9 | 4.2 | 4.28 | 0.44 | 1.3 |
| | 2 | 16.3 | 4.2 | 4.15 | 0.41 | 1.2 |
| | 3 | 14 | 3.5 | 4.17 | 0.41 | 1.0 |
| | **average** | **15.4** | **4.0** | **4.2** | **0.42** | **1.2** |

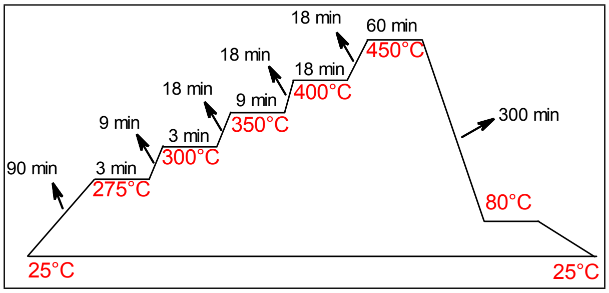


**Figure S12.** Sintering temperature program used for the fabrication of the 0.5 cm$^2$ ZnO working electrodes.

## References SI.


(1) Godfroy, M.; Liotier, J.; Mwalukuku, V. M.; Joly, D.; Huaulmé, Q.; Cabau, L.; Aumaitre, C.; Kervella, Y.; Narbey, S.; Oswald, F.; Palomares, E.; González Flores, C. A.; Oskam, G.; Demadrille, R. Benzothiadiazole-Based Photosensitizers for Efficient and Stable Dye-Sensitized Solar Cells and 8.7% Efficiency Semi-Transparent Mini-Modules. *Sustain. Energ. Fuels* **2021**, *5*, 144–153.

(2) Canto-Aguilar, E.J. Rodriguez-Perez, M.; Garcia-Rodriguez, R.; Lizama-Tzec, F. I.; de Denko, A. T.; Osterloh, F. E.; Oskam, G. ZnO-Based Dye-Sensitized Solar Cells: Effects of Redox Couple and Dye Aggregation. *Eelectrochimica Acta.* **2017**, *258*, 396–404.

(3) Garcia-Rodriguez, R.; Jiang, R.; Canto-Aguilar, E. J.; Oskam, G.; Boschloo, G. Improving the Mass Transport of Copper-Complex Redox Mediators in Dye-Sensitized Solar Cells by Reducing the Inter-Electrode Distance. *Phys. Chem. Chem Phys.* **2017**, *19* (47), 32132–32142.
